# Effects of Vitamin C, Rosmarinic Acid, or Quercetin on Fertilisation-Related Gene Expression in Porcine Cumulus–Oocyte Complexes During In Vitro Maturation

**DOI:** 10.3390/ijms27093801

**Published:** 2026-04-24

**Authors:** Oana-Maria Boldura, Simona Marc, Călin Mircu, Ioan Huțu, Daiana Cocoș, Dorin Alexandru Vizitiu, Jelena Savici

**Affiliations:** Faculty of Veterinary Medicine, University of Life Sciences “King Mihai I” from Timisoara, Calea Aradului 119, 300645 Timisoara, Romania; oanaboldura@usvt.ro (O.-M.B.); calinmircu@usvt.ro (C.M.); ioanhutu@usvt.ro (I.H.); daiana.cocos.fmv@usvt.ro (D.C.); dorin.vizitiu@usvt.ro (D.A.V.)

**Keywords:** in vitro maturation (IVM), porcine cumulus–oocyte complexes, antioxidants, vitamin C, rosmarinic acid, quercetin, gene expression, fertilisation-related genes, oocyte quality

## Abstract

Antioxidant supplementation during in vitro maturation (IVM) has been proposed as a strategy to influence transcriptional responses in oocytes and cumulus–oocyte complexes. In this study, we investigated whether vitamin C, rosmarinic acid, or quercetin influence the expression of key fertilisation-associated genes (*CD9*, *ITGA6*, *MFGE8*, *ZP2*, and *ZP3*) in porcine cumulus–oocyte complexes (COCs). COCs were classified into three intrinsic quality groups (I–III) and matured in the presence or absence of antioxidants. Gene expression was quantified by RT-qPCR and analysed using a two-way ANOVA model to assess the effects of COC quality and treatment. *ZP2* and *ZP3* transcript levels were consistently lower in class II and III COCs than in class I controls (*p* < 0.001). Antioxidant supplementation was associated with treatment- and quality-dependent differences in gene expression. Quercetin was associated with the most pronounced upregulation, with Q I increasing *ZP2* expression to 2.95-fold and *ZP3* to 2.43-fold relative to class I controls (*p* < 0.001). Vitamin C was also associated with increased transcript abundance across several treatment groups, including class II and class III COCs, whereas rosmarinic acid exhibited more moderate and gene-specific effects. In contrast, *MFGE8* expression, which was elevated in lower-quality COCs, was reduced in antioxidant-treated class II and III complexes. These findings provide transcript-level evidence that antioxidant exposure during IVM is associated with treatment- and quality-dependent changes in fertilisation-related gene expression in porcine COCs.

## 1. Introduction

In vitro maturation (IVM) of porcine oocytes is frequently associated with culture-induced oxidative stress (OS), which has been linked to impaired coordination between nuclear and cytoplasmic maturation and, consequently, to reduced fertilisation rates and embryonic development [1,2,3]. Elevated reactive oxygen species (ROS) produced during standard IVM conditions compromise mitochondrial function, spindle integrity, and secretory pathway fidelity. This is especially detrimental to the zona pellucida (ZP)—the oocyte’s extracellular coat and the initial site of sperm binding [4,5,6]. Even when oocytes reach metaphase II, oxidative modifications in ZP ligand synthesis and presentation may result in a morphologically normal but functionally impaired fertilisation interface, molecularly altered at the level of gene expression and biosynthetic pathways, potentially compromising downstream fertilisation events.

In pigs, the ZP is primarily composed of *zona pellucida glycoprotein 2 (ZP2)*, *zona pellucida glycoprotein 3 (ZP3)*, and *zona pellucida glycoprotein 4 (ZP4)*, which are synthesised by the oocyte and processed via the ER–Golgi pathway before being assembled into a fibrillar matrix at the oolemma. Although *ZP4* is an important structural component of the zona pellucida, it was not included in the present analysis, as this study focused on genes more directly associated with sperm binding dynamics and fertilisation-related molecular interactions during in vitro maturation [6]. *ZP2* forms the main sperm-binding domain, facilitating high-affinity docking post-capacitation, while *ZP3* provides secondary binding signals and acrosome reaction-mediating glycans. Proper disulfide bond formation and glycosylation are thus essential for species-specific sperm adhesion [7,8]. Oxidative stress can suppress *ZP* genes transcription, disrupt ER protein folding and Golgi glycosylation, and trigger premature activation of cortical granule proteases that cleave *ZP2*. These alterations diminish the density and quality of ligands at the sperm–ZP interface [6,9].

Because successful fertilisation requires coordinated zona pellucida recognition and membrane fusion, our study evaluates a five-gene panel encompassing both interfaces: *ZP2* and *ZP3* (zona pellucida recognition), *CD9* and *ITGA6* (oolemma adhesion and fusion competence), and *MFGE8/SED1* (a bridging ligand with EGF-like and discoidin domains). This panel enables a sequential assessment of key molecular events associated with fertilisation under in vitro maturation conditions [10,11,12].

At the fertilisation interface, our molecular analysis focused on the zona pellucida genes *ZP2* and *ZP3*. The N-terminal domain of *ZP2* critically determines homologous sperm binding, and its cleavage after fertilisation is key to preventing polyspermy [13,14]. Monitoring *ZP2* transcript levels during IVM, particularly under antioxidant supplementation, provides an indicator of whether culture conditions maintain the biosynthesis of the primary sperm-binding landscape. *ZP3*, meanwhile, displays specific N-linked glycans that support *ZP2*-mediated adhesion and regulate acrosome exocytosis. Proper glycosylation of *ZP3* requires an intact ER/Golgi redox environment and functional enzymatic machinery [1,15,16]. As these glycan signals are especially susceptible to oxidative disruption during IVM, *ZP3* expression serves as a marker for glycan-dependent ligand quality at the *ZP* surface.

To couple zona recognition to the membrane fusion step, we included *CD9*, *ITGA6*, and *MFGE8/SED1*, which together report the oolemma’s adhesion/fusion competence and its molecular bridging to the ZP. *CD9* is a tetraspanin that organises tetraspanin–integrin microdomains required for sperm–oocyte membrane fusion; its abundance and distribution are shaped by the maturation media [17,18]. Assessing *CD9* enables evaluation of whether oxidative control during IVM maintains a fusion-competent oolemma. *ITGA6*, typically pairing as α6β1/α6β4, contributes to adhesion and signalling at the fusion interface and is implicated in gamete membrane interactions and early developmental processes [7,19], providing an adhesion axis complementary to *CD9*. *MFGE8/SED1*, featuring EGF-like and discoidin domains, interacts with sperm and zona components and supports cell–matrix communication in the peri-ovulatory environment [10,20,21]. In this context, *MFGE8* may serve as a molecular mediator bridging sperm, the ZP, and the oolemma. Collectively, this five-gene panel covers extracellular recognition (*ZP2*, *ZP3*), membrane-level adhesion and fusion (*CD9*, *ITGA6*), and bridging/extracellular matrix support (*MFGE8*), providing a coherent framework for assessing fertilisation-related molecular processes during in vitro maturation. This approach allows evaluation of whether antioxidant supplementation preserves key structures involved in sperm–oocyte interaction under oxidative conditions [22,23].

Targeted antioxidants were selected to provide complementary protection across aqueous and membrane compartments and along the transcription–folding–glycosylation–secretion axis. Vitamin C (ascorbic acid) offers water-soluble ROS scavenging and can support glutathione-dependent folding conditions and redox-sensitive transcription during early IVM [24,25]. Rosmarinic acid, an amphipathic phenolic, attenuates oxidative injury and can induce endogenous antioxidants, thereby helping protect ER/Golgi enzymology relevant to *ZP3* glycan maturation [26,27,28]. Quercetin combines direct radical quenching with mitochondrial stabilisation and has improved porcine IVM outcomes in several reports, including reduced intracellular ROS and enhanced developmental competence [29,30,31,32]. Broad experience with small-molecule antioxidants in oocyte culture indicates dose- and context-dependence and suggests that transcriptional programs governing extracellular matrix synthesis are responsive to redox modulation [33,34,35,36].

In this study, we investigated whether supplementation of porcine IVM media with vitamin C, rosmarinic acid, or quercetin is associated with differences in the transcript abundance of selected fertilisation-related genes in cumulus–oocyte complexes of different morphological quality classes. The analysis focused on *ZP2* and *ZP3* as markers related to zona pellucida structure and sperm-binding functions, on *CD9* and *ITGA6* in relation to membrane adhesion and fusion-associated processes, and on *MFGE8* as a gene linked to bridging and extracellular matrix-related interactions. The objective was to determine whether antioxidant supplementation, applied at concentrations selected from previous porcine IVM studies, is associated with treatment- and quality-dependent transcriptional changes in this gene panel during in vitro maturation [3,25,37].

## 2. Results

**Figure 1:**
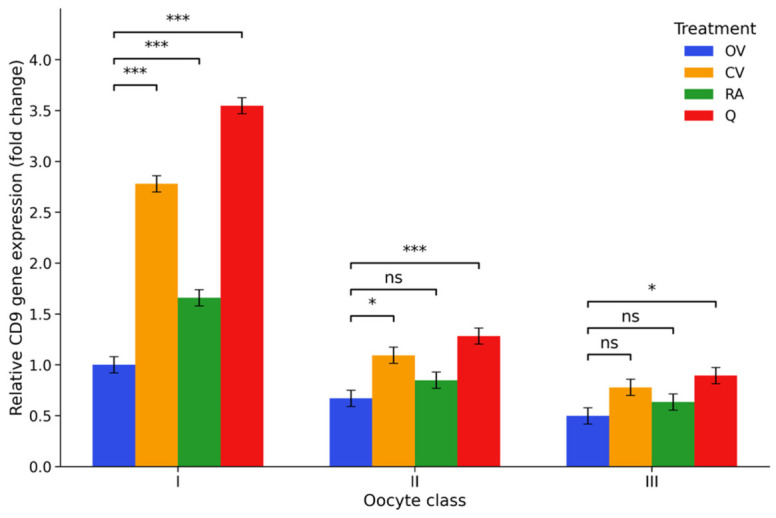
*CD9* gene expression under antioxidant supplementation. Relative CD9 mRNA expression (2^−ΔΔCt^, fold change) in porcine cumulus–oocyte complexes (COCs) following in vitro maturation. Expression levels were normalised to the reference gene PPIA and expressed relative to the OV I group, which was set to 1. Experimental groups included control medium (OV I–III), vitamin C-supplemented medium (CV I–III), rosmarinic acid-supplemented medium (RA I–III), and quercetin-supplemented medium (Q I–III), applied to oocytes classified according to morphological quality: class I (high-quality COCs), class II (intermediate-quality COCs), and class III (low-quality COCs). Data are presented as estimated marginal means ± 95% confidence interval (*n* = 6 biological replicates). Statistical analysis was performed using two-way ANOVA (factors: COC class and treatment) followed by Tukey’s post hoc test for multiple comparisons. Significance brackets indicate comparisons between the control and antioxidant-treated groups within each morphological class. * *p* < 0.05, *** *p* < 0.001; ns, not significant.

**Figure 2:**
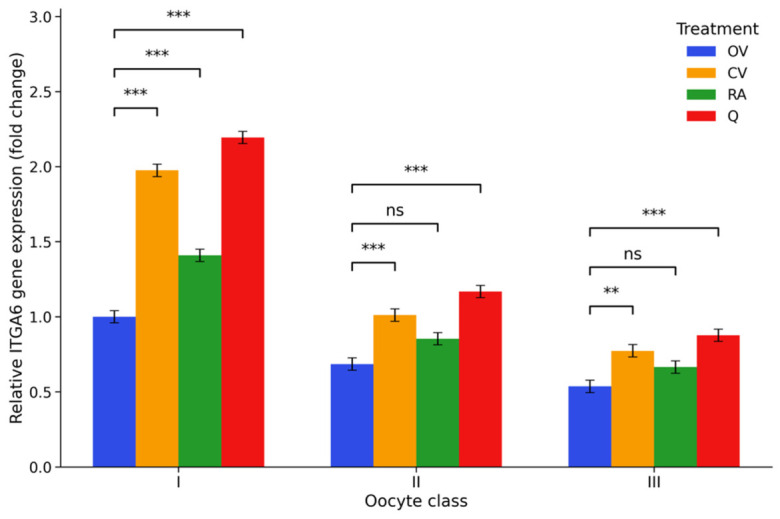
ITGA6 gene expression under antioxidant supplementation. Relative ITGA6 mRNA levels (2^−ΔΔCt^, fold change) in porcine cumulus–oocyte complexes following in vitro maturation. Fold changes are expressed relative to the OV I group, which was set to 1. Experimental groups included control medium (OV I–III), vitamin C-supplemented medium (CV I–III), rosmarinic acid-supplemented medium (RA I–III), and quercetin-supplemented medium (Q I–III), applied to COCs classified into morphological classes I–III, where class I represents high-quality oocytes, class II intermediate-quality oocytes, and class III low-quality oocytes. Values represent estimated marginal means (EMMs) ± 95% CI (*n* = 6). Statistical analysis was performed using two-way ANOVA followed by Tukey’s post hoc test for multiple comparisons. Significance brackets indicate comparisons between the control and antioxidant-treated groups within each morphological class. ** *p* < 0.01, *** *p* < 0.001; ns, not significant.

**Figure 3:**
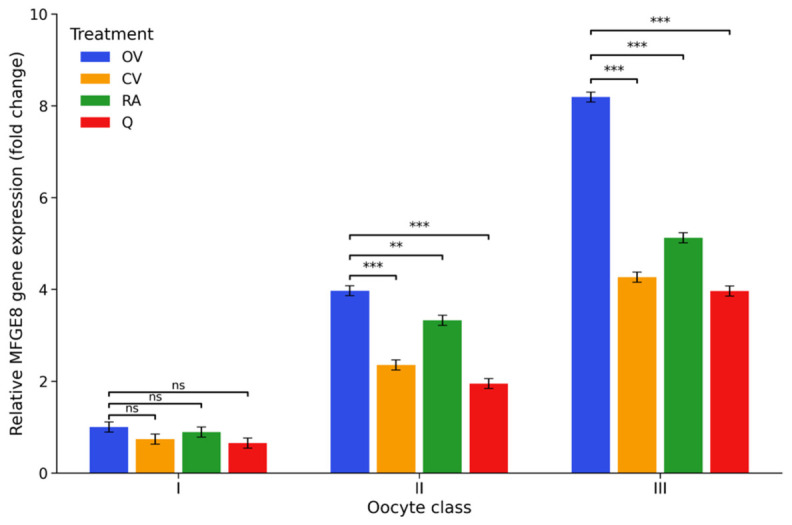
*MFGE8* gene expression under antioxidant supplementation. Relative MFGE8 mRNA levels (2^−ΔΔCt^, fold change) in porcine cumulus–oocyte complexes following in vitro maturation. Fold changes are expressed relative to the OV I group, which was set to 1. Experimental groups included control medium (OV I–III), vitamin C-supplemented medium (CV I–III), rosmarinic acid-supplemented medium (RA I–III), and quercetin-supplemented medium (Q I–III), applied to oocytes of morphological classes I–III, where class I represents high-quality oocytes, class II intermediate-quality oocytes, and class III low-quality oocytes. Values represent estimated marginal means (EMMs) ± 95% CI (*n* = 6). Statistical analysis was performed using two-way ANOVA (COC class × treatment) followed by Tukey’s post hoc test for multiple comparisons. Significance brackets indicate comparisons between the control and antioxidant-treated groups within each morphological class. ** *p* < 0.01, *** *p* < 0.001; ns, not significant.

**Figure 4:**
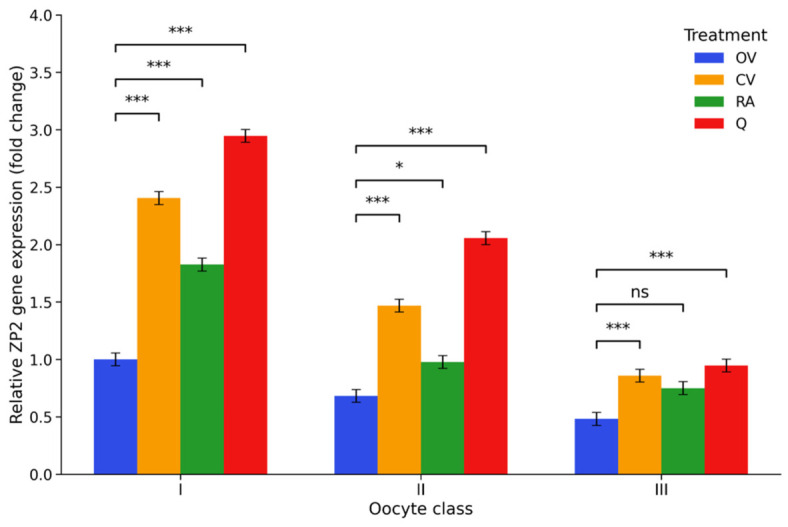
*ZP2* gene expression under antioxidant supplementation. Relative *ZP2* mRNA levels (2^−ΔΔCt^, fold change) in porcine cumulus–oocyte complexes following in vitro maturation. Fold changes are expressed relative to the OV I group, which was set to 1. Experimental groups included control medium (OV I–III), vitamin C-supplemented medium (CV I–III), rosmarinic acid-supplemented medium (RA I–III), and quercetin-supplemented medium (Q I–III), applied to oocytes of morphological classes I–III, where class I represents high-quality oocytes, class II intermediate-quality oocytes, and class III low-quality oocytes. Values represent estimated marginal means (EMMs) ± 95% CI (*n* = 6). Statistical analysis was performed using two-way ANOVA (COC class × treatment) followed by Tukey’s post hoc test for multiple comparisons. Significance brackets indicate comparisons between the control and antioxidant-treated groups within each morphological class. * *p* < 0.05, *** *p* < 0.001; ns, not significant.

**Figure 5:**
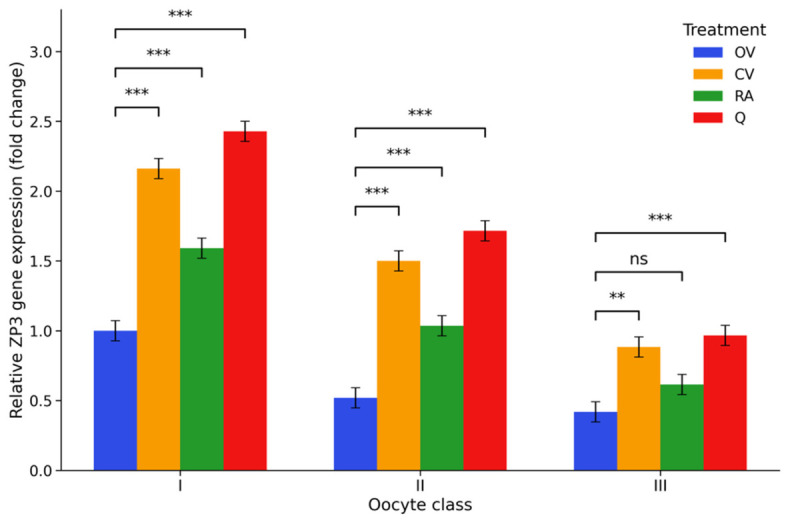
Relative *ZP3* mRNA levels (2^−ΔΔCt^, fold change) in porcine cumulus–oocyte complexes following in vitro maturation. Fold changes are expressed relative to the OV I group, which was set to 1. Experimental groups included control medium (OV I–III), vitamin C-supplemented medium (CV I–III), rosmarinic acid-supplemented medium (RA I–III), and quercetin-supplemented medium (Q I–III), applied to oocytes of morphological classes I–III, where class I represents high-quality oocytes, class II intermediate-quality oocytes, and class III low-quality oocytes. Values represent estimated marginal means (EMMs) ± 95% CI (*n* = 6). Statistical analysis was performed using two-way ANOVA (COC class × treatment) followed by Tukey’s post hoc test for multiple comparisons. Significance brackets indicate comparisons between the control and antioxidant-treated groups within each morphological class. ** *p* < 0.01, *** *p* < 0.001; ns, not significant.

To evaluate whether fertilisation-associated transcript abundance during porcine in vitro maturation is modulated by antioxidant supplementation, the relative expression of five target genes—*CD9*, *ITGA6*, *MFGE8*, *ZP2*, and *ZP3*—was quantified in cumulus–oocyte complexes (COCs) using the 2^−ΔΔCt^ method. COCs were stratified into three intrinsic morphological quality classes: OV I (class I, high-quality control COCs), OV II (class II, intermediate-quality COCs), and OV III (class III, poor-quality COCs typically excluded from IVF procedures). Additional experimental groups were created from COCs of each quality class matured in media supplemented with vitamin C (CV I–III), rosmarinic acid (RA I–III), or quercetin (Q I–III). Highly significant effects of COC quality class, antioxidant treatment, and their interaction were revealed by statistical analysis using two-way ANOVA across all analysed genes (*p* < 0.001). Significant differences in gene expression depending on both treatment condition and COC quality class were indicated by post hoc comparisons based on Tukey’s test. The results are detailed below and illustrated in Figure 1, Figure 2, Figure 3, Figure 4 and Figure 5; the corresponding full two-way ANOVA outputs and post hoc comparisons are provided in the Supplementary Materials (Supplementary File S1).

*CD9* transcript abundance varied significantly depending on both the intrinsic morphological quality of the cumulus–oocyte complexes (COCs) and the antioxidant treatment applied during in vitro maturation. In the control groups, *CD9* expression progressively declined with decreasing COC quality. Relative transcript levels were highest in class I complexes (OV I, 1.00-fold) and were significantly lower in class II (OV II, 0.67-fold) and class III COCs (OV III, 0.50-fold) (*p* < 0.001), indicating a marked reduction in this fusion-associated transcript in morphologically compromised complexes.

Antioxidant supplementation altered this pattern in a treatment-dependent manner. Vitamin C supplementation markedly increased *CD9* transcript abundance in class I COCs (CV I, 2.78-fold), representing a highly significant increase compared with the class I control group (*p* < 0.001). A more moderate elevation was observed in class II complexes (CV II, 1.09-fold), while expression in class III COCs (CV III, 0.78-fold) remained below the baseline level observed in class I controls.

Rosmarinic acid produced a weaker stimulatory effect on *CD9* expression. In class I COCs, RA I resulted in a moderate increase (1.66-fold), whereas transcript levels remained comparatively low in class II and class III complexes (RA II, 0.85-fold; RA III, 0.63-fold). In contrast, quercetin induced the strongest transcriptional response among the tested antioxidants. *CD9* expression in class I COCs increased to 3.55-fold in the Q I group, representing the highest level observed across all treatments (*p* < 0.001 vs. OV I). In class II and class III complexes, quercetin also elevated transcript abundance relative to the corresponding controls (Q II, 1.28-fold; Q III, 0.89-fold), although expression remained lower than that observed in class I antioxidant-treated groups.

Two-way ANOVA confirmed highly significant effects of COC class, treatment, and their interaction on *CD9* expression (*p* < 0.001). Post hoc comparisons further demonstrated significant differences between control and antioxidant-treated groups, particularly for vitamin C and quercetin in class I complexes, while several comparisons among treatments in lower-quality COCs showed smaller or non-significant differences. Overall, the data reveal a clear interaction between intrinsic COC quality and antioxidant supplementation in determining CD9 transcript abundance during in vitro maturation, as illustrated in Figure 1; detailed statistical outputs are provided in Supplementary File S1.

*ITGA6* transcript abundance showed a clear dependence on COC quality, with progressively lower expression observed from class I to class III COCs. Compared with class I controls, transcript levels were significantly reduced in both class II and class III complexes (*p* < 0.001), indicating a gradual decline in this adhesion-related marker as morphological quality decreased. Antioxidant supplementation altered this pattern to varying degrees. Vitamin C produced a marked increase in *ITGA6* expression, particularly in class I COCs, where transcript levels reached approximately 1.98-fold relative to the control group (*p* < 0.001). A comparable but slightly stronger effect was observed following quercetin supplementation, with the Q I group displaying the highest transcript abundance among all treatments (2.19-fold; *p* < 0.001). Rosmarinic acid also elevated *ITGA6* expression in class I complexes, although the magnitude of the increase was more moderate (1.41-fold; *p* = 0.001). In the lower-quality classes, antioxidant supplementation resulted in more modest changes. While vitamin C and quercetin maintained higher transcript levels than those detected in the corresponding control groups, the increases observed after rosmarinic acid supplementation in class II and class III COCs did not reach statistical significance. Overall, the data indicate that antioxidant supplementation was associated with higher ITGA6 transcript levels in several treatment groups, with quercetin showing the strongest effect. The distribution of *ITGA6* transcript levels across treatments and COC classes is illustrated in Figure 2, while the corresponding pairwise comparisons and full statistical outputs are presented in Supplementary File S1.

*MFGE8* displayed a distinct expression pattern compared with the other analysed genes. Rather than decreasing with declining COC quality, *MFGE8* transcript abundance increased markedly across the COC classes. Relative to class I controls, expression levels rose sharply in class II and class III complexes, reaching approximately 3.97- and 8.19-fold, respectively (*p* < 0.001), indicating a pronounced upregulation of *MFGE8* in morphologically compromised COCs. Antioxidant supplementation modified this pattern in a class-dependent manner. In class I COCs, *MFGE8* transcript levels were numerically lower in all antioxidant-treated groups than in the untreated control group (CV I: 0.74-fold; RA I: 0.89-fold; Q I: 0.65-fold), but these differences were not statistically significant. In class II COCs, all three antioxidants significantly reduced *MFGE8* expression relative to the corresponding control group, with the lowest mean value observed after quercetin supplementation (Q II: 1.95-fold), followed by vitamin C (CV II: 2.35-fold) and rosmarinic acid (RA II: 3.33-fold). In class III COCs, *MFGE8* expression also remained significantly lower in all antioxidant-treated groups than in the untreated control group, although transcript abundance remained markedly elevated relative to class I. Overall, these findings indicate that antioxidant supplementation attenuates the *MFGE8* overexpression associated with reduced COC quality, but does not restore expression to the baseline level observed in class I control COCs. The distribution of *MFGE8* transcript levels across the experimental groups is illustrated in Figure 3; the complete statistical results supporting these comparisons are provided in Supplementary File S1.

*ZP2* transcript abundance declined progressively with decreasing COC quality. Compared with class I control cumulus–oocyte complexes (COCs), expression levels were significantly reduced in class II and class III complexes, reaching approximately 0.68- and 0.48-fold of control values, respectively (*p* < 0.001), indicating compromised *ZP2* transcription in morphologically inferior COCs. Antioxidant supplementation markedly modified this pattern. Vitamin C treatment substantially increased *ZP2* expression across all classes, with transcript levels exceeding those of class I controls in class I complexes and remaining higher than the corresponding untreated groups in classes II and III. Rosmarinic acid produced a more moderate response, with RA I increasing transcript abundance to approximately 1.83-fold, whereas RA II and RA III remained closer to the values observed in untreated class II and class III COCs. Among the tested antioxidants, quercetin exerted the strongest effect, significantly increasing *ZP2* expression in class I and class II complexes (2.95- and 2.06-fold, respectively) and also increasing transcript levels in class III COCs. These findings indicate that antioxidant supplementation was associated with higher ZP2 transcript abundance across several treatment groups despite the reduced expression observed in lower-quality COCs. The distribution of *ZP2* expression levels across the experimental groups is presented in Figure 4, with the corresponding full statistical outputs provided in Supplementary File S1.

*ZP3* transcript abundance declined markedly with decreasing COC quality. Compared with class I control cumulus–oocyte complexes (COCs), expression levels were significantly reduced in class II and class III complexes, reaching approximately 0.52- and 0.42-fold of control values, respectively (*p* < 0.001), indicating impaired *ZP3* transcription in morphologically compromised COCs. Antioxidant supplementation substantially modified this pattern. Vitamin C treatment significantly increased *ZP3* expression across all COC classes, elevating transcript levels to approximately 2.16-fold in class I complexes and to levels above those observed in the corresponding untreated groups in classes II and III. Rosmarinic acid produced a more moderate response, with RA I increasing transcript abundance to approximately 1.59-fold, whereas RA II and RA III remained closer to the levels observed in untreated class II and class III COCs. Among the tested antioxidants, quercetin exerted the strongest effect, markedly increasing *ZP3* expression in class I and class II complexes (2.43- and 1.72-fold, respectively) and increasing transcript levels in class III COCs. Overall, these findings indicate that antioxidant supplementation was associated with higher *ZP3* transcript abundance across several treatment groups, despite lower expression in reduced-quality COCs. The distribution of *ZP3* transcript levels among the experimental groups is presented in Figure 5, and the corresponding detailed statistical outputs are available in Supplementary File S1.

Taken together, these results show that antioxidant supplementation was associated with treatment- and gene-dependent differences in transcript abundance across porcine cumulus–oocyte complexes of varying morphological quality. Across the analysed genes, class II and class III complexes generally displayed expression patterns distinct from those of class I controls, and antioxidant exposure modified these patterns to varying extents. Vitamin C and quercetin were associated with the largest shifts in transcript abundance across several genes, whereas rosmarinic acid generally produced more moderate and gene-dependent changes. These findings indicate that antioxidant supplementation was associated with transcriptional alterations observed in COCs of reduced morphological quality, with effects that varied according to the analysed gene and the treatment applied during in vitro maturation.

## 3. Discussion

Establishing effective in vitro maturation (IVM) and in vitro fertilisation (IVF) protocols remains essential for porcine reproductive biotechnology and for the pig’s role as a translational model in human reproductive biology. Owing to their genetic and physiological similarities to humans, pigs are widely used to study fertilisation, embryogenesis, and assisted reproduction. In this context, the quality and molecular integrity of porcine cumulus–oocyte complexes (COCs) during IVM are critical determinants of downstream fertilisation-related processes [9,34]. Nevertheless, porcine IVF continues to be affected by persistent challenges, including high polyspermy rates, variable developmental competence of cumulus–oocyte complexes, and the susceptibility of gametes to culture-induced stress [3,9,38]. Addressing these limitations requires improved culture systems that preserve the molecular integrity of cumulus–oocyte complexes and maintain the expression of critical transcripts and pathways associated with fertilisation-related processes during IVM.

Oxidative stress is widely recognised as an important factor affecting the success of IVM and IVF, as the accumulation of reactive oxygen species in vitro leads to mitochondrial dysfunction, lipid peroxidation, DNA damage, and altered gene expression, which have been associated with reduced fertilisation rates and impaired embryonic development [36,39]. A range of antioxidant interventions has been explored, from classical agents such as vitamin C, N-acetylcysteine (NAC), and glutathione donors to plant-derived polyphenols, including resveratrol, melatonin, and flavonoids [40,41,42,43]. These compounds have been reported to act through multiple mechanisms, including direct neutralisation of reactive oxygen species, restoration of intracellular glutathione levels, activation of NRF2-mediated antioxidant pathways, and stabilisation of mitochondrial function [44,45]. Nonetheless, the efficacy of these strategies remains inconsistent across studies, likely reflecting differences in antioxidant type, concentration, timing of application, and species-specific responses [25,46].

Against this backdrop, vitamin C, rosmarinic acid, and quercetin were selected for supplementation during porcine IVM. Vitamin C, used as a reference antioxidant, is a well-characterised reducing agent with recognised reactive oxygen species scavenging properties and a role in glutathione-dependent redox cycling [12,47]. Rosmarinic acid, chosen for its amphipathic polyphenolic structure, has been reported to localise to cellular membranes, where it may help limit lipid peroxidation and promote NRF2-dependent antioxidant responses [48]. Quercetin, a flavonoid with potent free radical scavenging and anti-apoptotic activities, was included based on evidence of its role in preserving mitochondrial integrity and modulating apoptosis-related genes in oocytes [31,49]. By evaluating these agents in parallel, we aimed to compare the transcriptional responses to these antioxidant treatments during porcine IVM and to examine whether plant-derived polyphenols elicited responses similar to those observed with vitamin C. Similar antioxidant-induced changes in gene transcription during in vitro maturation of porcine cumulus–oocyte complexes have been reported previously, particularly for genes involved in oxidative stress responses and developmental competence, supporting the transcriptional sensitivity of COCs to redox modulation [25,47,49].

Our findings revealed distinct gene-specific responses to antioxidant supplementation. *ZP2* and *ZP3*, which encode zona pellucida glycoproteins involved in sperm binding and acrosome reaction-related processes, were significantly downregulated in class II and class III COCs. It should be noted that gene expression was assessed in whole cumulus–oocyte complexes. While zona pellucida-related genes such as *ZP2* and *ZP3* are predominantly expressed by the oocyte, other analysed genes (*CD9*, *ITGA6*, and *MFGE8*) may also be expressed in cumulus cells. Therefore, the observed transcriptional profiles likely reflect predominantly oocyte-derived expression, while not excluding potential contributions from the surrounding cumulus compartment. This pattern is consistent with previous reports showing that oxidative stress can affect zona pellucida-related molecular features, including glycosylation-associated processes involved in sperm–oocyte recognition [5,6,9]. In the present study, quercetin and vitamin C were associated with higher ZP2 and ZP3 transcript levels in lower-quality cumulus–oocyte complexes, in some cases exceeding those observed in class I control COCs. These findings suggest that antioxidant supplementation may be associated with modulation of zona pellucida-related transcriptional profiles in COCs of reduced morphological quality during IVM [34].

*CD9* and *ITGA6*, both involved in oolemma-associated fusion-related processes, were also downregulated in lower-quality cumulus–oocyte complexes. *CD9*, a tetraspanin involved in organising membrane microdomains required for sperm–egg fusion, and *ITGA6*, an integrin subunit implicated in adhesion to cumulus cells and extracellular matrix components, have both been linked to cellular responses that may be affected under oxidative conditions [12,36]. In the present study, quercetin and vitamin C were associated with higher CD9 and ITGA6 transcript levels in class III cumulus–oocyte complexes, reaching values comparable to those observed in class I controls. These findings suggest that antioxidant supplementation may be associated with modulation of fusion-related transcriptional profiles in lower-quality COCs during IVM [31,35]. Rosmarinic acid exerted a more limited effect, possibly reflecting a narrower effective dose range under the experimental conditions used [26].

*MFGE8* showed a distinct expression pattern compared with the other genes analysed, as its transcript abundance increased rather than decreased in lower-quality COCs. Unlike *ZP2*, *ZP3*, *CD9*, or *ITGA6*, for which reduced expression in poorer-quality complexes is more readily interpreted as loss of fertilisation-associated molecular features, *MFGE8* appears to follow a different transcriptional pattern [19]. Its elevated expression in class II and class III COCs may reflect altered cellular conditions within the cumulus–oocyte complex, potentially in relation to changes in extracellular organisation, integrin-associated signalling, or broader homeostatic responses [50,51]. Similar increases in *MFGE8* expression have also been reported in stress-associated cellular conditions [50], which is consistent with the possibility that the higher transcript levels observed here reflect a compensatory response to a compromised intracellular environment. In this context, the lower *MFGE8* transcript levels observed after antioxidant supplementation, particularly in the poorer-quality classes, are more appropriately interpreted as attenuation of an altered transcriptional profile than as enhancement of a competence-related marker [52,53]. This pattern is compatible with the proposed role of antioxidant supplementation in modulating redox-sensitive signalling pathways and cellular homeostasis under oxidative conditions [53]. Because the present study assessed transcript abundance only, without protein-level or functional validation, these observations should not be interpreted as evidence of restored *MFGE8* function. Rather, they indicate that antioxidant supplementation was associated with modulation of stress-related or dysregulated transcriptional responses during IVM, without fully re-establishing the expression pattern observed in class I control COCs [28,54].

**Figure 6:**
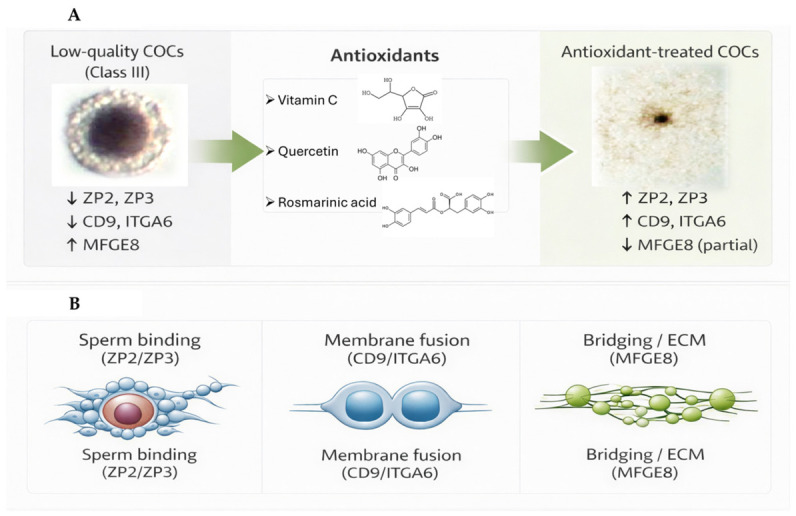
Conceptual summary of the transcriptional changes associated with antioxidant supplementation during porcine COCs in vitro maturation. (**A**): Schematic representation of the gene expression profile observed in low-quality cumulus–oocyte complexes (COCs; class III) and of the overall transcriptional changes associated with antioxidant supplementation during in vitro maturation. Low-quality COCs showed lower transcript levels of *ZP2*, *ZP3*, *CD9*, and *ITGA6*, together with higher *MFGE8* expression. Antioxidant treatment was associated with increased expression of *ZP2*, *ZP3*, *CD9*, and *ITGA6*, and a partial reduction in *MFGE8* overexpression. Arrows indicate the schematic direction of the observed transcriptional changes, with upward arrows representing increased transcript abundance and downward arrows representing decreased transcript abundance. (**B**): Schematic functional grouping of the analysed genes in relation to fertilisation-associated processes. *ZP2* and *ZP3* are linked to sperm binding at the zona pellucida, CD9 and ITGA6 to membrane fusion-related events, and *MFGE8* to extracellular bridging/extracellular matrix interactions.

An additional point arising from these findings is that class III cumulus–oocyte complexes, which are generally excluded from IVF procedures because of their poor morphological quality, displayed pronounced transcriptional alterations across multiple fertilisation-associated genes. In the present study, antioxidant supplementation, particularly with quercetin and vitamin C, was associated with higher transcript levels for several of these genes, in some cases approaching those observed in class I complexes. These observations suggest that antioxidant treatment may influence the transcriptional profiles of COCs considered morphologically suboptimal, although the biological significance of these changes remains to be clarified by functional studies [55,56]. To facilitate the integrated interpretation of these gene-specific responses, a schematic overview of the main findings is presented in Figure 6.

A limitation of the present study is that fertilisation competence was inferred from transcriptional and morphological indicators rather than assessed through direct functional outcomes. While the analysed genes are well-established markers of zona pellucida integrity, membrane fusion competence, and sperm–oocyte interaction, changes in mRNA abundance do not necessarily correspond to equivalent changes at the protein levels or successful fertilisation. Accordingly, the transcriptional differences observed after antioxidant supplementation should be interpreted as molecular observations relevant to fertilisation-related processes, rather than as direct evidence of improved fertilisation or embryo developmental success. Future studies incorporating IVF assays, polyspermy assessment, and embryo development endpoints will be important for clarifying the functional significance of these findings.

Taken together, these findings indicate that antioxidant supplementation is associated with changes in the expression of fertilisation-related genes in porcine COCs. Across the analysed gene panel, vitamin C, rosmarinic acid, and quercetin were associated with treatment-dependent differences in transcripts related to zona pellucida structure, membrane fusion-associated processes, and extracellular signalling during in vitro maturation. Because the present study did not assess protein abundance, post-translational modifications, or zona pellucida functionality, these observations should be interpreted as transcript-level findings rather than as evidence of demonstrated mechanistic or functional effects. The differences observed among morphological quality classes nevertheless highlight the value of stratifying cumulus–oocyte complexes in IVF-related research and support further investigation of antioxidant supplementation in relation to COC quality [40,47,48,57].

## 4. Materials and Methods

### 4.1. Biological Material and Ovary Collection

Porcine ovaries were obtained from a commercial local abattoir immediately after slaughter, placed in 0.9% NaCl supplemented with PenStrep (100×; Gibco, Thermo Fisher Scientific, Waltham, MA, USA; Cat. No. 15140122) and transported in insulated containers at ~32 °C to the Assisted Reproduction Laboratory (USVT, “Horia Cernescu” Laboratory Complex) within ~30 min. No live-animal procedures were performed. Upon arrival, ovaries were rinsed in warmed Dulbecco’s PBS (Biowest, Nuaillé, France)and processed for COC retrieval.

### 4.2. Retrieval and Classification of Cumulus–Oocyte Complexes (COCs)

Follicles of 1–5 mm diameter were aspirated using a 5 mL Luer-Lock syringe (BD, Franklin Lakes, NJ, USA) fitted with an 18-gauge needle. Follicular fluid was pooled in conical tubes containing PBS and allowed to sediment; COCs were identified under a stereomicroscope (Stemi 2000-C, Carl Zeiss, Jena, Germany) with hote plate (ThermoPlate, Mats-UST2, Tokai Hit, Fujinomiya-shi, Japan) and washed. Following collection, cumulus–oocyte complexes (COCs) were examined under a stereomicroscope and morphologically classified according to established criteria based on cumulus cell investment and ooplasm appearance, as previously described by Jackowska et al. (2009) [58], and further applied and validated in porcine IVM studies, including our previous work [25,59].

Briefly, class I COCs were characterised by a homogeneous, translucent ooplasm and a complete, compact cumulus oophorus composed of multiple tightly packed cell layers. Class II COCs exhibited a generally homogeneous cytoplasm with an incomplete but compact cumulus investment. Class III COCs displayed a heterogeneous or darkened ooplasm and a poorly compacted, dispersed, or partially absent cumulus oophorus, features consistently associated with reduced developmental competence.

This morphological classification has been shown to reliably reflect COC quality and maturation potential in porcine IVM systems and was therefore used in the present study to stratify oocytes before antioxidant supplementation and downstream molecular analyses.

### 4.3. COC Handling and Pre-IVM Media

During ovary processing and COC selection, Dulbecco-PBS (500 mL; Biowest, Nuaillé, France) was freshly supplemented (per 500 mL) with glucose (500 mg; Sigma-Aldrich, St. Louis, MO, USA, sodium pyruvate (18 mg; P3662, Sigma-Aldrich, St. Louis, MO, USA) penicillin (10 mg; Sigma-Aldrich, St. Louis, MO, USA) streptomycin (20 mg; Sigma-Aldrich, St. Louis, MO, USA), heparin (5.6 mg; Sigma-Aldrich, St. Louis, MO, USA), and BSA (150 mg; Sigma-Aldrich, St. Louis, MO, USA), and maintained at 35 °C on IVF heating plate (Minitube HT400, Minitüb GmbH, Tiefenbach, Germany) [38,60,61].

### 4.4. Antioxidants and Study Design

Three antioxidants were evaluated during in vitro maturation (*IVM*): vitamin C (L-ascorbic acid, CAS 50-81-7; Calbiochem, Merck, Darmstadt, Germany), prepared in ultrapure water, 0.5 mM final concentration; rosmarinic acid (CAS 20283-92-5; Sigma-Aldrich, St. Louis, MO, USA), prepared in DMSO (D8418, Sigma-Aldrich, St. Louis, MO, USA), 105 µM final concentration; quercetin dihydrate (CAS 6151-25-3; Calbiochem, Merck, Darmstadt, Germany), prepared in DMSO, 10 µM final concentration. The selected concentrations were based on previous studies in porcine oocyte culture systems reporting antioxidant efficacy within defined ranges that avoid cytotoxic or pro-oxidant effects. All compounds were used within previously validated concentration ranges for oocyte maturation systems (vitamin C: 0.1–1 mM; rosmarinic acid: 50–200 µM; quercetin: 5–20 µM). The concentrations used in the present study fall within these reported ranges and have been previously shown to be compatible with oocyte maturation and developmental competence [25,29,31].

Fresh working solutions were prepared immediately prior to use and were not stored for prolonged periods. In the case of vitamin C, this approach was specifically adopted to minimise oxidation and ensure compound stability during the experimental procedure. All antioxidant solutions were added directly to the maturation medium after preparation. For all antioxidant treatments, the final DMSO concentration in the maturation medium did not exceed 0.1% (*v*/*v*), a level widely reported as non-toxic in porcine cumulus–oocyte complex culture systems [29,31].

Cumulus–oocyte complexes (COCs) were collected during two independent ovary collection sessions and processed separately. Within each session, COCs were classified by morphological quality and allocated to control or antioxidant-supplemented maturation conditions. For each treatment × quality-class combination, independent pooled COC samples were generated for downstream RNA extraction. Biological replication was therefore based on independently prepared pooled COC samples obtained from separate collection and processing events.

COCs from each morphological class (I–III) were distributed into experimental groups as follows: control medium (OV I–III), vitamin C-supplemented medium (CV I–III), rosmarinic acid-supplemented medium (RA I–III), and quercetin-supplemented medium (Q I–III), with a fixed antioxidant concentration applied across all morphological classes within each treatment group.

This design allowed assessment of antioxidant effects within each baseline quality class.

### 4.5. In Vitro Maturation (IVM)

IVM was performed in TCM-199 (HEPES-modified, M2520; Sigma-Aldrich, St. Louis, MO, USA) supplemented with NaHCO_3_ (220 mg/100 mL, S6014; Sigma-Aldrich, St. Louis, MO, USA), gentamicin (5 mg/100 mL, G1914; Sigma-Aldrich, St. Louis, MO, USA), and sodium pyruvate (2.2 mg/100 mL). Prior to use, the maturation medium was equilibrated for ≥4 h at 38.5 °C in 5% CO_2_ maximal humidity), ensuring adequate temperature and pH stabilisation before the introduction of cumulus–oocyte complexes. For maturation, COCs were placed in 400 µL drops of TCM-199 supplemented with follicle-stimulating hormone (FSH; final concentration 0.88 IU/mL; F8174, Sigma-Aldrich, St. Louis, MO, USA) and overlaid with mineral oil in 4-well plates. Incubation was for 44 h at 38.5 °C, 5% CO_2_ in a CO_2_ incubator (MCO-18AC, Sakata, Japan). After IVM, cumulus expansion was assessed under a stereomicroscope with a heated stage [33]. All COCs included in downstream analyses were those that completed the in vitro maturation period and met morphological criteria for post-IVM viability. Morphological classification of cumulus–oocyte complexes (COCs) and in vitro maturation (IVM) outcomes for the experimental groups analysed in this study have been previously reported in detail [59]. For this reason, these parameters are not presented again here, as the current study is focused on transcriptomic analysis.

### 4.6. Target Genes and Primer Design

Gene expression was focused on *CD9*, *ITGA6*, *MFGE8*, *ZP2*, *ZP3* genes, and *peptidylprolyl isomerase A (PPIA)*, with *PPIA* serving as the reference gene.

For targets lacking validated porcine assays, de novo primer pairs were designed in silico: protein sequences were identified (UniProt/NCBI), mapped to porcine mRNA via tblastn, and cDNA sequences were used to design primers with NCBI’s “pick primers” tool. Newly designed oligonucleotides were synthesised by Eurogentec (Seraing, Belgium). For newly designed primer pairs, experimental validation was performed prior to RT-qPCR by endpoint PCR using cDNA as template, followed by agarose gel electrophoresis to confirm a single amplicon of the expected size and to exclude nonspecific products or primer-dimer formation. The primer sequences are listed in Table 1.

### 4.7. RNA Extraction and cDNA Synthesis

Total RNA was extracted from pooled whole cumulus–oocyte complexes (COCs) for each experimental group. Each biological replicate consisted of an independent pooled COC sample corresponding to one treatment × quality-class combination. For each biological replicate, pooled material obtained from approximately 15–25 COCs (corresponding to ~1.5 × 10^3^ cells per sample) was processed separately for RNA isolation. RNA extraction was performed using the SV Total RNA Isolation System (Promega, Madison, WI, USA) according to the manufacturer’s protocol. A total of six biological replicates (*n* = 6) were analysed, generated from material collected across two independent oocyte collection sessions. Gene expression analysis was performed for each biological replicate in technical triplicate, and mean Ct values were used for subsequent calculations. RNA purity and concentration were assessed on a NanoDrop™ 8000 spectrophotometer (Thermo Fisher Scientific, Waltham, MA, USA) (A260/280 ≈ 1.8; A260/230 ≈ 2.0). Complementary DNA (cDNA) was synthesised using SuperScript™ IV VILO™ Master Mix (Invitrogen™, Thermo Fisher Scientific, Waltham, MA, USA) in 20 µL reaction volumes: 10 µL RNA + 10 µL master mix.

### 4.8. RT-qPCR

Quantitative PCR used GoTaq^®^ qPCR Master Mix (Promega; SYBR Green chemistry with ROX passive dye) on an ABI 7500 Real-Time PCR System. Each reaction (20 µL) contained 10 µL 2× Master Mix, 0.4 µL forward primer (10 µM), 0.4 µL reverse primer (10 µM), cDNA (equivalent to ~200 ng RNA input), and nuclease-free water. Each biological replicate consisted of an independent pool of cumulus–oocyte complexes (COCs) obtained from separate ovary collection sessions. qPCR measurements were performed in technical triplicate. Amplification was performed using the instrument’s default cycling protocol, with an annealing temperature of 60 °C for all assays. Specificity of amplification was further verified by melt-curve analysis after each run, confirming the presence of a single peak for each assay. No-template controls (NTC) were included for every gene. Primer performance was assessed during assay optimisation by confirming amplification specificity and an acceptable efficiency range of 90–110%. Expression was normalised to *PPIA* and calculated using the 2^−^^ΔΔCt^ method [64].

### 4.9. Statistical Analysis

qPCR gene expression data were analysed in Jamovi (v2.6; The Jamovi Project, 2024), using R (v4.4; R Core Team, 2024) as the computational backend. Before statistical testing, the data were examined in Jamovi to assess distributional assumptions and for potential outliers. The datasets were considered suitable for ANOVA, and no data points were excluded from the analysis. A two-way analysis of variance (ANOVA) was then used to evaluate the effects of oocyte morphological class and antioxidant treatment, as well as their interaction. When significant effects were identified, pairwise comparisons were performed using Tukey’s post hoc test based on estimated marginal means. Differences were considered statistically significant at *p* < 0.05. The unit of biological replication was an independent pooled RNA preparation obtained from cumulus–oocyte complexes (COCs). Graphical representations of the data were generated in Microsoft Excel using estimated marginal means and statistical comparisons derived from Jamovi. Full statistical outputs, including two-way ANOVA tables and post hoc comparisons, are provided in the Supplementary Materials (Supplementary File S1).

## 5. Conclusions

Antioxidant supplementation during porcine in vitro maturation was associated with modulation of the expression of selected fertilisation-associated genes in cumulus–oocyte complexes in a manner dependent on both COCs’ quality and treatment type. A consistent reduction in the expression of zona pellucida- and fusion-related genes (*ZP2*, *ZP3*, *CD9*, and *ITGA6*) was observed in lower-quality COCs, whereas antioxidant exposure altered this pattern to varying extents. Vitamin C and quercetin were associated with the largest transcriptional changes, particularly in class III complexes, where they were linked to increased transcript levels of these genes, while rosmarinic acid exerted a more moderate and gene-specific influence, including attenuation of the altered *MFGE8* expression pattern.

These findings indicate that antioxidant supplementation can partially modulate transcriptional profiles associated with reduced COC quality, although the extent and direction of the response varied among the analysed genes. The differential responses observed among the tested compounds further suggest that their effects depend on both the intrinsic characteristics of the oocyte and the specific molecular targets evaluated in this study.

While these results provide insight into transcriptional changes under antioxidant-supplemented IVM conditions, further studies are required to determine whether these molecular alterations are accompanied by corresponding changes in fertilisation outcomes and subsequent embryonic development.

## Figures and Tables

**Table 1:** Primer sequences, amplicon sizes, and accession numbers for genes analysed by qPCR.

Gene	Accession No. (NCBI)	Primer Sequence (5′→3′)	Amplicon Size (bp)	Primer Source
CD9	NM_214006.1	F: CTCATGATGGTGGTGGGCTT R: GGAATATCCCCAGATGGCCG	98	Original source
ITGA6	XM_021076079	F: ATGAAGGGGAAGGAGGACGA R: CAAAGCCGCAATGACACA	122	Original source
MFGE8	NM_001122984	F: GCCTTCTCCGGTGACTTCT R: GGGCGTCATCAATCACCTCA	121	Original source
ZP2	NM_213848.2	F: GCGGTTTGGTCAGCAATGAG R: TGTCCCGGCTTGCCATAAAT	108	Original source
ZP3	NM_213893.1	F: TCACCGTGGATGTGTTCCAT R: GGCTTTGTTGAGTTGGTCCG	110	Original source
PPIA *	NM_214353.1	F: AAAACTTCCGTGCTCTGAGC R: TTATGGCGTGTGAAGTCACC	119	Ref. [62], validated in [63]

* Primer sequences were adopted from the cited sources. PPIA was selected as the endogenous control based on our previous validation study.

## Data Availability

The original contributions presented in this study are included in the article/Supplementary Material. Further inquiries can be directed to the corresponding authors.

## Supplementary Materials

The following supporting information can be downloaded at: https://www.mdpi.com/article/10.3390/ijms27093801/s1.

## Abbreviations

The following abbreviations are used in this manuscript:

IVM	in vitro maturation
OS	oxidative stress
ROS	reactive oxygen species
ZP	zona pellucida
ECM	extracellular matrix
COCs	cumulus–oocyte complexes
NTC	no-template controls
IVF	in vitro fertilisation
NAC	N-acetylcysteine
