# Supplementary material for: Effects of Vitamin C, Rosmarinic Acid, or Quercetin on Fertilisation-Related Gene Expression in Porcine Cumulus–Oocyte Complexes During In Vitro Maturation
Source: Int J Mol Sci. 2026 Apr 24;27(9):3801. doi: 10.3390/ijms27093801 (PMC13164473; doi:10.3390/ijms27093801)
Supplement: Supplementary file 1 [file ijms-27-03801-s001.zip › ijms-4189968-supplementary.pdf]

Results

ANOVA

ANOVA - CD9 gene expression (x fold)

	Sum of Squares	df	Mean Square	F	p
**oocyte class**	32.67	2	16.3325	428.2	<.001
**treatment**	14.93	3	4.9751	130.4	<.001
**oocyte class ∗ treatment**	10.15	6	1.6923	44.4	<.001
**Residuals**	2.29	60	0.0381		

[3]

Post Hoc Tests

Post Hoc Comparisons - oocyte class

Comparison							
**oocyte class**		**oocyte class**	**Mean Difference**	**SE**	**df**	**t**	p_{tukey}
**I**	-	**II**	1.273	0.0564	60.0	22.58	<.001
	-	**III**	1.545	0.0564	60.0	27.41	<.001
**II**	-	**III**	0.272	0.0564	60.0	4.83	<.001

Note. Comparisons are based on estimated marginal means

Post Hoc Comparisons - treatment

Comparison							
**treatment**		**treatment**	**Mean Difference**	**SE**	**df**	**t**	p_{tukey}
**OV**	-	**CV**	-0.828	0.0651	60.0	-12.72	<.001
	-	**RA**	-0.325	0.0651	60.0	-4.99	<.001
	-	**Q**	-1.185	0.0651	60.0	-18.21	<.001
**CV**	-	**RA**	0.503	0.0651	60.0	7.73	<.001
	-	**Q**	-0.357	0.0651	60.0	-5.49	<.001
**RA**	-	**Q**	-0.861	0.0651	60.0	-13.22	<.001

Note. Comparisons are based on estimated marginal means

Post Hoc Comparisons - oocyte class ✻ treatment

Comparison									
oocyte class	treatment		oocyte class	treatment	Mean Difference	SE	df	t	p_{tukey}
I	OV	-	I	CV	-1.7791	0.113	60.0	-15.779	<.001
		-	I	RA	-0.6589	0.113	60.0	-5.843	<.001
		-	I	Q	-2.5467	0.113	60.0	-22.587	<.001
		-	II	OV	0.3302	0.113	60.0	2.928	0.157
		-	II	CV	-0.0928	0.113	60.0	-0.823	1.000
		-	II	RA	0.1522	0.113	60.0	1.350	0.968
		-	II	Q	-0.2821	0.113	60.0	-2.502	0.359
		-	III	OV	0.5031	0.113	60.0	4.462	0.002
		-	III	CV	0.2218	0.113	60.0	1.967	0.713
		-	III	RA	0.3658	0.113	60.0	3.244	0.075
		-	III	Q	0.1064	0.113	60.0	0.944	0.998
	CV	-	I	RA	1.1203	0.113	60.0	9.936	<.001
		-	I	Q	-0.7675	0.113	60.0	-6.807	<.001
		-	II	OV	2.1093	0.113	60.0	18.708	<.001
		-	II	CV	1.6863	0.113	60.0	14.956	<.001
		-	II	RA	1.9314	0.113	60.0	17.130	<.001
		-	II	Q	1.4971	0.113	60.0	13.278	<.001
		-	III	OV	2.2822	0.113	60.0	20.241	<.001
		-	III	CV	2.0009	0.113	60.0	17.746	<.001
		-	III	RA	2.1449	0.113	60.0	19.024	<.001
		-	III	Q	1.8855	0.113	60.0	16.723	<.001
	RA	-	I	Q	-1.8878	0.113	60.0	-16.743	<.001
		-	II	OV	0.9890	0.113	60.0	8.772	<.001
		-	II	CV	0.5660	0.113	60.0	5.020	<.001
		-	II	RA	0.8111	0.113	60.0	7.194	<.001
		-	II	Q	0.3768	0.113	60.0	3.342	0.058
		-	III	OV	1.1619	0.113	60.0	10.305	<.001
		-	III	CV	0.8806	0.113	60.0	7.810	<.001
		-	III	RA	1.0246	0.113	60.0	9.088	<.001
		-	III	Q	0.7652	0.113	60.0	6.787	<.001
	Q	-	II	OV	2.8768	0.113	60.0	25.515	<.001
		-	II	CV	2.4538	0.113	60.0	21.763	<.001
		-	II	RA	2.6989	0.113	60.0	23.937	<.001
		-	II	Q	2.2646	0.113	60.0	20.085	<.001
		-	III	OV	3.0497	0.113	60.0	27.048	<.001
		-	III	CV	2.7684	0.113	60.0	24.554	<.001
		-	III	RA	2.9124	0.113	60.0	25.831	<.001
		-	III	Q	2.6530	0.113	60.0	23.530	<.001

Note. Comparisons are based on estimated marginal means

Post Hoc Comparisons - oocyte class ✻ treatment

**II**	**OV**	-	**II**	**CV**	-0.4230	0.113	60.0	-3.752	0.019
		-	**II**	**RA**	-0.1779	0.113	60.0	-1.578	0.910
		-	**II**	**Q**	-0.6123	0.113	60.0	-5.430	<.001
		-	**III**	**OV**	0.1729	0.113	60.0	1.533	0.925
		-	**III**	**CV**	-0.1084	0.113	60.0	-0.962	0.998
		-	**III**	**RA**	0.0356	0.113	60.0	0.316	1.000
		-	**III**	**Q**	-0.2238	0.113	60.0	-1.985	0.701
	**CV**	-	**II**	**RA**	0.2451	0.113	60.0	2.174	0.574
		-	**II**	**Q**	-0.1892	0.113	60.0	-1.678	0.871
		-	**III**	**OV**	0.5959	0.113	60.0	5.285	<.001
		-	**III**	**CV**	0.3146	0.113	60.0	2.790	0.211
		-	**III**	**RA**	0.4586	0.113	60.0	4.068	0.007
		-	**III**	**Q**	0.1992	0.113	60.0	1.767	0.829
	**RA**	-	**II**	**Q**	-0.4343	0.113	60.0	-3.852	0.014
		-	**III**	**OV**	0.3508	0.113	60.0	3.111	0.104
		-	**III**	**CV**	0.0695	0.113	60.0	0.617	1.000
		-	**III**	**RA**	0.2135	0.113	60.0	1.894	0.758
		-	**III**	**Q**	-0.0459	0.113	60.0	-0.407	1.000
	**Q**	-	**III**	**OV**	0.7851	0.113	60.0	6.964	<.001
		-	**III**	**CV**	0.5038	0.113	60.0	4.469	0.002
		-	**III**	**RA**	0.6479	0.113	60.0	5.746	<.001
		-	**III**	**Q**	0.3885	0.113	60.0	3.445	0.044
**III**	**OV**	-	**III**	**CV**	-0.2813	0.113	60.0	-2.495	0.363
		-	**III**	**RA**	-0.1373	0.113	60.0	-1.217	0.985
		-	**III**	**Q**	-0.3967	0.113	60.0	-3.518	0.036
	**CV**	-	**III**	**RA**	0.1440	0.113	60.0	1.277	0.979
		-	**III**	**Q**	-0.1154	0.113	60.0	-1.023	0.997
	**RA**	-	**III**	**Q**	-0.2594	0.113	60.0	-2.301	0.487

Note. Comparisons are based on estimated marginal means

[4]

Estimated Marginal Means

oocyte class ✻ treatment

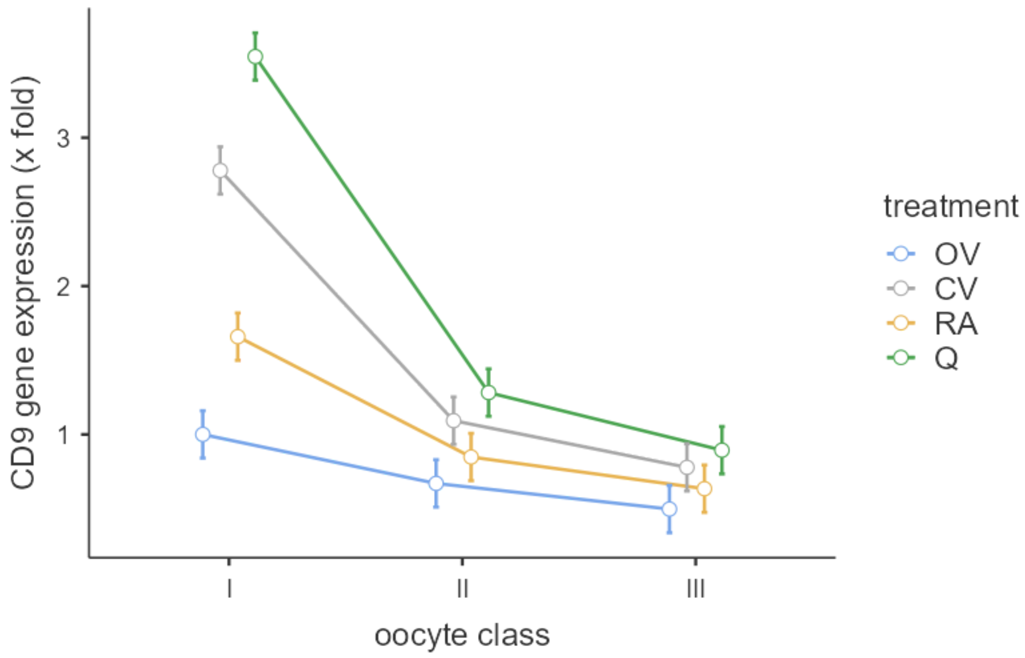

Estimated Marginal Means - oocyte class ✻ treatment

treatment	oocyte class	Mean	SE	95% Confidence Interval	
				Lower	Upper
**OV**	**I**	1.000	0.0797	0.841	1.159
	**II**	0.670	0.0797	0.510	0.829
	**III**	0.497	0.0797	0.337	0.656
**CV**	**I**	2.779	0.0797	2.620	2.939
	**II**	1.093	0.0797	0.933	1.252
	**III**	0.778	0.0797	0.619	0.938
**RA**	**I**	1.659	0.0797	1.499	1.818
	**II**	0.848	0.0797	0.688	1.007
	**III**	0.634	0.0797	0.475	0.794
**Q**	**I**	3.547	0.0797	3.387	3.706
	**II**	1.282	0.0797	1.123	1.442
	**III**	0.894	0.0797	0.734	1.053

Results

ANOVA

ANOVA - ITGA6 gene expression (x fold)

	Sum of Squares	df	Mean Square	F	p
**oocyte class**	11.408	2	5.7038	569.6	<.001
**treatment**	4.784	3	1.5948	159.3	<.001
**oocyte class ∗ treatment**	1.664	6	0.2773	27.7	<.001
**Residuals**	0.601	60	0.0100		

[3]

Post Hoc Tests

Post Hoc Comparisons - oocyte class

Comparison							
**oocyte class**		**oocyte class**	**Mean Difference**	**SE**	**df**	**t**	p_{tukey}
**I**	-	**II**	0.715	0.0289	60.0	24.75	<.001
	-	**III**	0.932	0.0289	60.0	32.25	<.001
**II**	-	**III**	0.217	0.0289	60.0	7.50	<.001

Note. Comparisons are based on estimated marginal means

Post Hoc Comparisons - treatment

Comparison							
**treatment**		**treatment**	**Mean Difference**	**SE**	**df**	**t**	p_{tukey}
**OV**	-	**CV**	-0.512	0.0334	60.0	-15.36	<.001
	-	**RA**	-0.236	0.0334	60.0	-7.06	<.001
	-	**Q**	-0.672	0.0334	60.0	-20.16	<.001
**CV**	-	**RA**	0.277	0.0334	60.0	8.30	<.001
	-	**Q**	-0.160	0.0334	60.0	-4.79	<.001
**RA**	-	**Q**	-0.437	0.0334	60.0	-13.09	<.001

Note. Comparisons are based on estimated marginal means

Post Hoc Comparisons - oocyte class ✻ treatment

Comparison									
oocyte class	treatment		oocyte class	treatment	Mean Difference	SE	df	t	p_{tukey}
**I**	**OV**	-	**I**	**CV**	-0.9751	0.0578	60.0	-16.879	<.001
		-	**I**	**RA**	-0.4088	0.0578	60.0	-7.077	<.001
		-	**I**	**Q**	-1.1938	0.0578	60.0	-20.664	<.001
		-	**II**	**OV**	0.3147	0.0578	60.0	5.446	<.001
		-	**II**	**CV**	-0.0108	0.0578	60.0	-0.186	1.000
		-	**II**	**RA**	0.1460	0.0578	60.0	2.527	0.344
		-	**II**	**Q**	-0.1680	0.0578	60.0	-2.907	0.165
		-	**III**	**OV**	0.4639	0.0578	60.0	8.029	<.001
		-	**III**	**CV**	0.2269	0.0578	60.0	3.928	0.011
		-	**III**	**RA**	0.3346	0.0578	60.0	5.791	<.001
		-	**III**	**Q**	0.1232	0.0578	60.0	2.133	0.602
	**CV**	-	**I**	**RA**	0.5663	0.0578	60.0	9.802	<.001
		-	**I**	**Q**	-0.2187	0.0578	60.0	-3.785	0.017
		-	**II**	**OV**	1.2898	0.0578	60.0	22.325	<.001
		-	**II**	**CV**	0.9644	0.0578	60.0	16.693	<.001
		-	**II**	**RA**	1.1211	0.0578	60.0	19.406	<.001
		-	**II**	**Q**	0.8072	0.0578	60.0	13.971	<.001
		-	**III**	**OV**	1.4390	0.0578	60.0	24.908	<.001
		-	**III**	**CV**	1.2021	0.0578	60.0	20.807	<.001
		-	**III**	**RA**	1.3097	0.0578	60.0	22.670	<.001
		-	**III**	**Q**	1.0984	0.0578	60.0	19.012	<.001
	**RA**	-	**I**	**Q**	-0.7850	0.0578	60.0	-13.587	<.001
		-	**II**	**OV**	0.7235	0.0578	60.0	12.523	<.001
		-	**II**	**CV**	0.3981	0.0578	60.0	6.890	<.001
		-	**II**	**RA**	0.5548	0.0578	60.0	9.604	<.001
		-	**II**	**Q**	0.2409	0.0578	60.0	4.169	0.005
		-	**III**	**OV**	0.8727	0.0578	60.0	15.106	<.001
		-	**III**	**CV**	0.6358	0.0578	60.0	11.005	<.001
		-	**III**	**RA**	0.7434	0.0578	60.0	12.867	<.001
		-	**III**	**Q**	0.5321	0.0578	60.0	9.210	<.001
	**Q**	-	**II**	**OV**	1.5085	0.0578	60.0	26.110	<.001
		-	**II**	**CV**	1.1831	0.0578	60.0	20.478	<.001
		-	**II**	**RA**	1.3398	0.0578	60.0	23.191	<.001
		-	**II**	**Q**	1.0258	0.0578	60.0	17.756	<.001
		-	**III**	**OV**	1.6577	0.0578	60.0	28.693	<.001
		-	**III**	**CV**	1.4207	0.0578	60.0	24.592	<.001
		-	**III**	**RA**	1.5284	0.0578	60.0	26.455	<.001
		-	**III**	**Q**	1.3170	0.0578	60.0	22.797	<.001

Note. Comparisons are based on estimated marginal means

Post Hoc Comparisons - oocyte class ✻ treatment

**II**	**OV**	**-**	**II**	**CV**	-0.3254	0.0578	60.0	-5.633	<.001
		**-**	**II**	**RA**	-0.1687	0.0578	60.0	-2.919	0.161
		**-**	**II**	**Q**	-0.4826	0.0578	60.0	-8.354	<.001
		**-**	**III**	**OV**	0.1492	0.0578	60.0	2.583	0.312
		**-**	**III**	**CV**	-0.0877	0.0578	60.0	-1.518	0.929
		**-**	**III**	**RA**	0.0199	0.0578	60.0	0.344	1.000
		**-**	**III**	**Q**	-0.1914	0.0578	60.0	-3.313	0.063
	**CV**	**-**	**II**	**RA**	0.1568	0.0578	60.0	2.713	0.245
		**-**	**II**	**Q**	-0.1572	0.0578	60.0	-2.721	0.241
		**-**	**III**	**OV**	0.4746	0.0578	60.0	8.215	<.001
		**-**	**III**	**CV**	0.2377	0.0578	60.0	4.114	0.006
		**-**	**III**	**RA**	0.3453	0.0578	60.0	5.977	<.001
		**-**	**III**	**Q**	0.1340	0.0578	60.0	2.319	0.475
	**RA**	**-**	**II**	**Q**	-0.3140	0.0578	60.0	-5.435	<.001
		**-**	**III**	**OV**	0.3179	0.0578	60.0	5.502	<.001
		**-**	**III**	**CV**	0.0809	0.0578	60.0	1.401	0.959
		**-**	**III**	**RA**	0.1885	0.0578	60.0	3.264	0.071
		**-**	**III**	**Q**	-0.0228	0.0578	60.0	-0.394	1.000
	**Q**	**-**	**III**	**OV**	0.6319	0.0578	60.0	10.937	<.001
		**-**	**III**	**CV**	0.3949	0.0578	60.0	6.835	<.001
		**-**	**III**	**RA**	0.5025	0.0578	60.0	8.698	<.001
		**-**	**III**	**Q**	0.2912	0.0578	60.0	5.041	<.001
**III**	**OV**	**-**	**III**	**CV**	-0.2370	0.0578	60.0	-4.101	0.006
		**-**	**III**	**RA**	-0.1293	0.0578	60.0	-2.238	0.530
		**-**	**III**	**Q**	-0.3406	0.0578	60.0	-5.896	<.001
	**CV**	**-**	**III**	**RA**	0.1076	0.0578	60.0	1.863	0.776
		**-**	**III**	**Q**	-0.1037	0.0578	60.0	-1.795	0.814
	**RA**	**-**	**III**	**Q**	-0.2113	0.0578	60.0	-3.658	0.025

Note. Comparisons are based on estimated marginal means

[4]

Estimated Marginal Means

oocyte class ✻ treatment

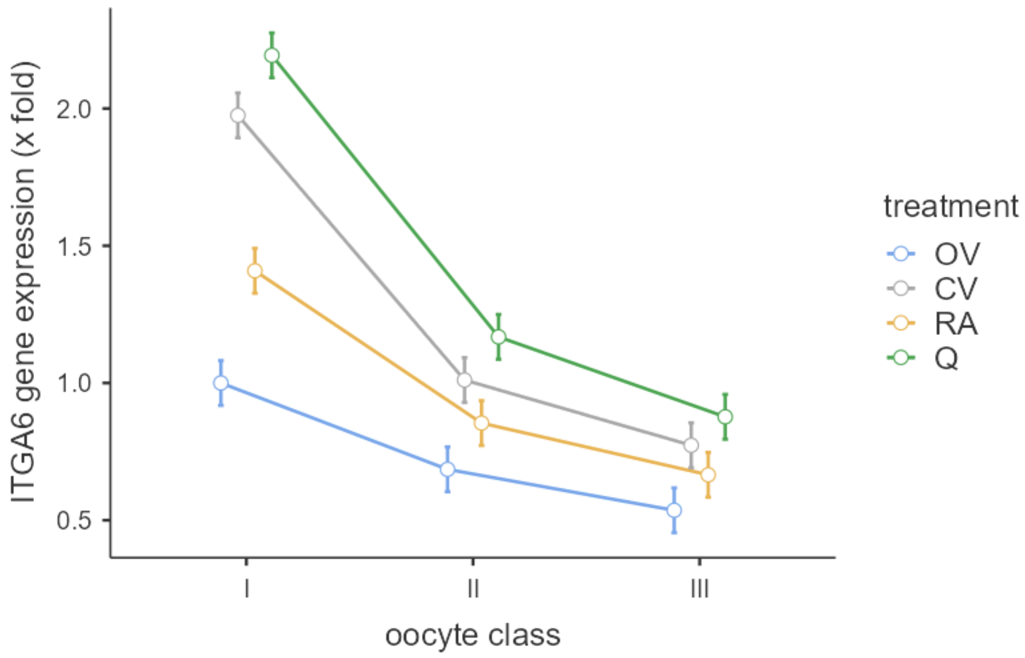

Estimated Marginal Means - oocyte class ✻ treatment

treatment	oocyte class	Mean	SE	95% Confidence Interval	
				Lower	Upper
**OV**	**I**	1.000	0.0409	0.918	1.082
	**II**	0.685	0.0409	0.604	0.767
	**III**	0.536	0.0409	0.454	0.618
**CV**	**I**	1.975	0.0409	1.893	2.057
	**II**	1.011	0.0409	0.929	1.092
	**III**	0.773	0.0409	0.691	0.855
**RA**	**I**	1.409	0.0409	1.327	1.491
	**II**	0.854	0.0409	0.772	0.936
	**III**	0.665	0.0409	0.584	0.747
**Q**	**I**	2.194	0.0409	2.112	2.276
	**II**	1.168	0.0409	1.086	1.250
	**III**	0.877	0.0409	0.795	0.958

Results

ANOVA

ANOVA - MFGE8 gene expression (x fold)

	Sum of Squares	df	Mean Square	F	p
**oocyte class**	250.73	2	125.3658	1755.4	<.001
**treatment**	52.07	3	17.3572	243.0	<.001
**oocyte class ∗ treatment**	30.85	6	5.1414	72.0	<.001
**Residuals**	4.29	60	0.0714		

[3]

Post Hoc Tests

Post Hoc Comparisons - oocyte class

**Comparison**							
**oocyte class**		**oocyte class**	**Mean Difference**	**SE**	**df**	**t**	p_{tukey}
**I**	-	**II**	-2.08	0.0771	60.0	-26.9	<.001
	-	**III**	-4.56	0.0771	60.0	-59.2	<.001
**II**	-	**III**	-2.49	0.0771	60.0	-32.2	<.001

Note. Comparisons are based on estimated marginal means

Post Hoc Comparisons - treatment

**Comparison**							
**treatment**		**treatment**	**Mean Difference**	**SE**	**df**	**t**	p_{tukey}
**OV**	-	**CV**	1.935	0.0891	60.0	21.73	<.001
	-	**RA**	1.273	0.0891	60.0	14.30	<.001
	-	**Q**	2.200	0.0891	60.0	24.69	<.001
**CV**	-	**RA**	-0.662	0.0891	60.0	-7.43	<.001
	-	**Q**	0.264	0.0891	60.0	2.97	0.022
**RA**	-	**Q**	0.926	0.0891	60.0	10.40	<.001

Note. Comparisons are based on estimated marginal means

Post Hoc Comparisons - oocyte class ✻ treatment

Comparison									
oocyte class	treatment		oocyte class	treatment	Mean Difference	SE	df	t	p_{tukey}
I	OV	-	I	CV	0.26443	0.154	60.0	1.7139	0.855
		-	I	RA	0.10859	0.154	60.0	0.7038	1.000
		-	I	Q	0.34925	0.154	60.0	2.2636	0.513
		-	II	OV	-2.96883	0.154	60.0	-19.2418	<.001
		-	II	CV	-1.35218	0.154	60.0	-8.7639	<.001
		-	II	RA	-2.32483	0.154	60.0	-15.0679	<.001
		-	II	Q	-0.94672	0.154	60.0	-6.1359	<.001
		-	III	OV	-7.18945	0.154	60.0	-46.5968	<.001
		-	III	CV	-3.26447	0.154	60.0	-21.1579	<.001
		-	III	RA	-4.12178	0.154	60.0	-26.7144	<.001
		-	III	Q	-2.96190	0.154	60.0	-19.1969	<.001
	CV	-	I	RA	-0.15585	0.154	60.0	-1.0101	0.997
		-	I	Q	0.08482	0.154	60.0	0.5497	1.000
		-	II	OV	-3.23327	0.154	60.0	-20.9557	<.001
		-	II	CV	-1.61662	0.154	60.0	-10.4777	<.001
		-	II	RA	-2.58927	0.154	60.0	-16.7818	<.001
		-	II	Q	-1.21115	0.154	60.0	-7.8498	<.001
		-	III	OV	-7.45388	0.154	60.0	-48.3107	<.001
		-	III	CV	-3.52890	0.154	60.0	-22.8718	<.001
		-	III	RA	-4.38622	0.154	60.0	-28.4283	<.001
		-	III	Q	-3.22633	0.154	60.0	-20.9108	<.001
	RA	-	I	Q	0.24066	0.154	60.0	1.5598	0.916
		-	II	OV	-3.07742	0.154	60.0	-19.9456	<.001
		-	II	CV	-1.46077	0.154	60.0	-9.4677	<.001
		-	II	RA	-2.43342	0.154	60.0	-15.7717	<.001
		-	II	Q	-1.05530	0.154	60.0	-6.8397	<.001
		-	III	OV	-7.29804	0.154	60.0	-47.3006	<.001
		-	III	CV	-3.37305	0.154	60.0	-21.8617	<.001
		-	III	RA	-4.23037	0.154	60.0	-27.4182	<.001
		-	III	Q	-3.07049	0.154	60.0	-19.9007	<.001
	Q	-	II	OV	-3.31808	0.154	60.0	-21.5054	<.001
		-	II	CV	-1.70143	0.154	60.0	-11.0275	<.001
		-	II	RA	-2.67408	0.154	60.0	-17.3315	<.001
		-	II	Q	-1.29597	0.154	60.0	-8.3995	<.001
		-	III	OV	-7.53870	0.154	60.0	-48.8604	<.001
		-	III	CV	-3.61372	0.154	60.0	-23.4215	<.001
		-	III	RA	-4.47103	0.154	60.0	-28.9780	<.001
		-	III	Q	-3.31115	0.154	60.0	-21.4605	<.001

Note. Comparisons are based on estimated marginal means

Post Hoc Comparisons - oocyte class ✻ treatment

**II**	**OV**	**-**	**II**	**CV**	1.61665	0.154	60.0	10.4780	<.001
		**-**	**II**	**RA**	0.64400	0.154	60.0	4.1739	0.005
		**-**	**II**	**Q**	2.02212	0.154	60.0	13.1059	<.001
		**-**	**III**	**OV**	-4.22062	0.154	60.0	-27.3550	<.001
		**-**	**III**	**CV**	-0.29563	0.154	60.0	-1.9161	0.745
		**-**	**III**	**RA**	-1.15295	0.154	60.0	-7.4726	<.001
		**-**	**III**	**Q**	0.00693	0.154	60.0	0.0449	1.000
	**CV**	**-**	**II**	**RA**	-0.97265	0.154	60.0	-6.3040	<.001
		**-**	**II**	**Q**	0.40547	0.154	60.0	2.6279	0.288
		**-**	**III**	**OV**	-5.83727	0.154	60.0	-37.8329	<.001
		**-**	**III**	**CV**	-1.91228	0.154	60.0	-12.3940	<.001
		**-**	**III**	**RA**	-2.76960	0.154	60.0	-17.9505	<.001
		**-**	**III**	**Q**	-1.60972	0.154	60.0	-10.4330	<.001
	**RA**	**-**	**II**	**Q**	1.37812	0.154	60.0	8.9320	<.001
		**-**	**III**	**OV**	-4.86462	0.154	60.0	-31.5289	<.001
		**-**	**III**	**CV**	-0.93963	0.154	60.0	-6.0900	<.001
		**-**	**III**	**RA**	-1.79695	0.154	60.0	-11.6465	<.001
		**-**	**III**	**Q**	-0.63707	0.154	60.0	-4.1290	0.006
	**Q**	**-**	**III**	**OV**	-6.24273	0.154	60.0	-40.4609	<.001
		**-**	**III**	**CV**	-2.31775	0.154	60.0	-15.0220	<.001
		**-**	**III**	**RA**	-3.17507	0.154	60.0	-20.5785	<.001
		**-**	**III**	**Q**	-2.01518	0.154	60.0	-13.0610	<.001
**III**	**OV**	**-**	**III**	**CV**	3.92498	0.154	60.0	25.4389	<.001
		**-**	**III**	**RA**	3.06767	0.154	60.0	19.8824	<.001
		**-**	**III**	**Q**	4.22755	0.154	60.0	27.3999	<.001
	**CV**	**-**	**III**	**RA**	-0.85732	0.154	60.0	-5.5565	<.001
		**-**	**III**	**Q**	0.30257	0.154	60.0	1.9610	0.717
	**RA**	**-**	**III**	**Q**	1.15988	0.154	60.0	7.5175	<.001

Note. Comparisons are based on estimated marginal means

[4]

Estimated Marginal Means

oocyte class ✻ treatment

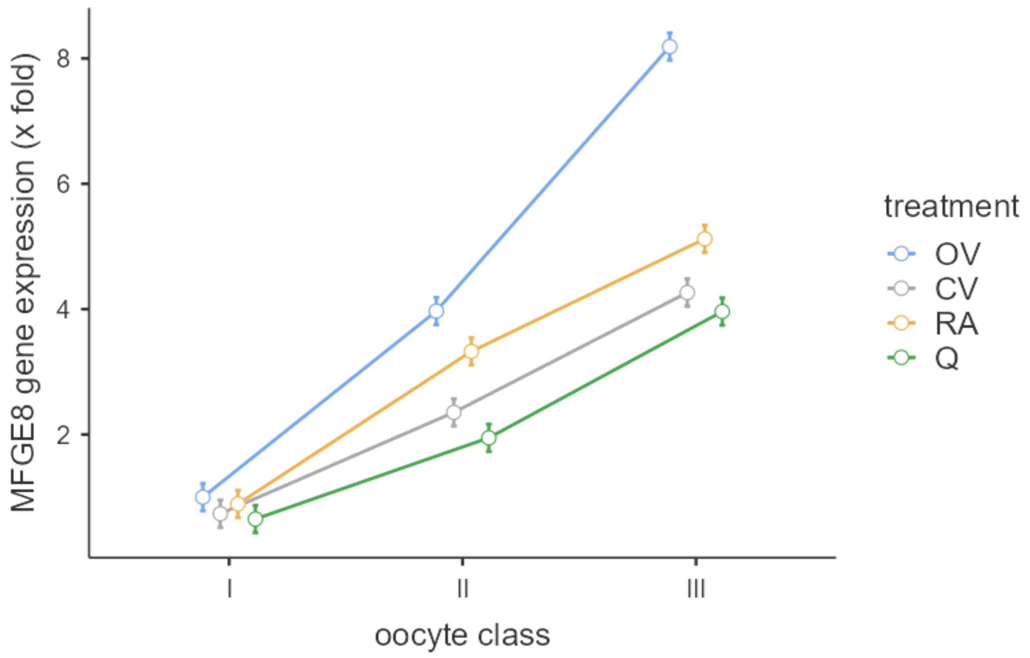

Estimated Marginal Means - oocyte class ✻ treatment

				95% Confidence Interval	
**treatment**	**oocyte class**	**Mean**	**SE**	**Lower**	**Upper**
**OV**	**I**	1.000	0.109	0.782	1.218
	**II**	3.969	0.109	3.751	4.187
	**III**	8.189	0.109	7.971	8.408
**CV**	**I**	0.736	0.109	0.517	0.954
	**II**	2.352	0.109	2.134	2.570
	**III**	4.264	0.109	4.046	4.483
**RA**	**I**	0.891	0.109	0.673	1.110
	**II**	3.325	0.109	3.107	3.543
	**III**	5.122	0.109	4.904	5.340
**Q**	**I**	0.651	0.109	0.433	0.869
	**II**	1.947	0.109	1.728	2.165
	**III**	3.962	0.109	3.744	4.180

Results

ANOVA

ANOVA - ZP2 gene expression (x fold)

	Sum of Squares	df	Mean Square	F	p
**oocyte class**	19.99	2	9.9932	531.2	<.001
**treatment**	15.73	3	5.2443	278.8	<.001
**oocyte class ✻ treatment**	4.00	6	0.6672	35.5	<.001
**Residuals**	1.13	60	0.0188		

[3]

Post Hoc Tests

Post Hoc Comparisons - oocyte class

Comparison							
**oocyte class**		**oocyte class**	**Mean Difference**	**SE**	**df**	**t**	**p_{tukey}**
**I**	-	**II**	0.748	0.0396	60.0	18.9	<.001
	-	**III**	1.285	0.0396	60.0	32.4	<.001
**II**	-	**III**	0.536	0.0396	60.0	13.5	<.001

Note. Comparisons are based on estimated marginal means

Post Hoc Comparisons - treatment

Comparison							
**treatment**		**treatment**	**Mean Difference**	**SE**	**df**	**t**	**p_{tukey}**
**OV**	-	**CV**	-0.856	0.0457	60.0	-18.72	<.001
	-	**RA**	-0.463	0.0457	60.0	-10.12	<.001
	-	**Q**	-1.262	0.0457	60.0	-27.60	<.001
**CV**	-	**RA**	0.393	0.0457	60.0	8.60	<.001
	-	**Q**	-0.406	0.0457	60.0	-8.88	<.001
**RA**	-	**Q**	-0.799	0.0457	60.0	-17.48	<.001

Note. Comparisons are based on estimated marginal means

Post Hoc Comparisons - oocyte class ✻ treatment

Comparison									
oocyte class	treatment		oocyte class	treatment	Mean Difference	SE	df	t	p_{tukey}
I	OV	-	I	CV	-1.4048	0.0792	60.0	-17.741	<.001
		-	I	RA	-0.8256	0.0792	60.0	-10.426	<.001
		-	I	Q	-1.9460	0.0792	60.0	-24.574	<.001
		-	II	OV	0.3175	0.0792	60.0	4.010	0.009
		-	II	CV	-0.4681	0.0792	60.0	-5.911	<.001
		-	II	RA	0.0233	0.0792	60.0	0.294	1.000
		-	II	Q	-1.0561	0.0792	60.0	-13.336	<.001
		-	III	OV	0.5183	0.0792	60.0	6.545	<.001
		-	III	CV	0.1417	0.0792	60.0	1.789	0.817
		-	III	RA	0.2500	0.0792	60.0	3.158	0.093
		-	III	Q	0.0525	0.0792	60.0	0.663	1.000
	CV	-	I	RA	0.5792	0.0792	60.0	7.314	<.001
		-	I	Q	-0.5411	0.0792	60.0	-6.834	<.001
		-	II	OV	1.7224	0.0792	60.0	21.750	<.001
		-	II	CV	0.9368	0.0792	60.0	11.830	<.001
		-	II	RA	1.4281	0.0792	60.0	18.035	<.001
		-	II	Q	0.3488	0.0792	60.0	4.405	0.002
		-	III	OV	1.9231	0.0792	60.0	24.286	<.001
		-	III	CV	1.5465	0.0792	60.0	19.530	<.001
		-	III	RA	1.6549	0.0792	60.0	20.898	<.001
		-	III	Q	1.4574	0.0792	60.0	18.404	<.001
	RA	-	I	Q	-1.1203	0.0792	60.0	-14.148	<.001
		-	II	OV	1.1432	0.0792	60.0	14.436	<.001
		-	II	CV	0.3575	0.0792	60.0	4.515	0.002
		-	II	RA	0.8489	0.0792	60.0	10.721	<.001
		-	II	Q	-0.2304	0.0792	60.0	-2.910	0.164
		-	III	OV	1.3439	0.0792	60.0	16.972	<.001
		-	III	CV	0.9673	0.0792	60.0	12.216	<.001
		-	III	RA	1.0757	0.0792	60.0	13.584	<.001
		-	III	Q	0.8782	0.0792	60.0	11.090	<.001
	Q	-	II	OV	2.2635	0.0792	60.0	28.584	<.001
		-	II	CV	1.4779	0.0792	60.0	18.663	<.001
		-	II	RA	1.9693	0.0792	60.0	24.868	<.001
		-	II	Q	0.8899	0.0792	60.0	11.238	<.001
		-	III	OV	2.4643	0.0792	60.0	31.119	<.001
		-	III	CV	2.0877	0.0792	60.0	26.364	<.001
		-	III	RA	2.1960	0.0792	60.0	27.732	<.001
		-	III	Q	1.9985	0.0792	60.0	25.238	<.001

Note. Comparisons are based on estimated marginal means

Post Hoc Comparisons - oocyte class ✻ treatment

**II**	**OV**	-	**II**	**CV**	-0.7856	0.0792	60.0	-9.921	<.001
		-	**II**	**RA**	-0.2942	0.0792	60.0	-3.715	0.021
		-	**II**	**Q**	-1.3736	0.0792	60.0	-17.346	<.001
		-	**III**	**OV**	0.2008	0.0792	60.0	2.536	0.339
		-	**III**	**CV**	-0.1758	0.0792	60.0	-2.220	0.542
		-	**III**	**RA**	-0.0675	0.0792	60.0	-0.852	0.999
		-	**III**	**Q**	-0.2650	0.0792	60.0	-3.346	0.058
	**CV**	-	**II**	**RA**	0.4914	0.0792	60.0	6.205	<.001
		-	**II**	**Q**	-0.5880	0.0792	60.0	-7.425	<.001
		-	**III**	**OV**	0.9864	0.0792	60.0	12.456	<.001
		-	**III**	**CV**	0.6098	0.0792	60.0	7.700	<.001
		-	**III**	**RA**	0.7181	0.0792	60.0	9.069	<.001
		-	**III**	**Q**	0.5206	0.0792	60.0	6.574	<.001
	**RA**	-	**II**	**Q**	-1.0793	0.0792	60.0	-13.630	<.001
		-	**III**	**OV**	0.4950	0.0792	60.0	6.251	<.001
		-	**III**	**CV**	0.1184	0.0792	60.0	1.495	0.936
		-	**III**	**RA**	0.2267	0.0792	60.0	2.863	0.181
		-	**III**	**Q**	0.0292	0.0792	60.0	0.369	1.000
	**Q**	-	**III**	**OV**	1.5744	0.0792	60.0	19.881	<.001
		-	**III**	**CV**	1.1977	0.0792	60.0	15.125	<.001
		-	**III**	**RA**	1.3061	0.0792	60.0	16.494	<.001
		-	**III**	**Q**	1.1086	0.0792	60.0	13.999	<.001
**III**	**OV**	-	**III**	**CV**	-0.3766	0.0792	60.0	-4.756	<.001
		-	**III**	**RA**	-0.2683	0.0792	60.0	-3.388	0.052
		-	**III**	**Q**	-0.4658	0.0792	60.0	-5.882	<.001
	**CV**	-	**III**	**RA**	0.1083	0.0792	60.0	1.368	0.965
		-	**III**	**Q**	-0.0892	0.0792	60.0	-1.126	0.992
	**RA**	-	**III**	**Q**	-0.1975	0.0792	60.0	-2.494	0.363

Note. Comparisons are based on estimated marginal means

[4]

Estimated Marginal Means

oocyte class ✻ treatment

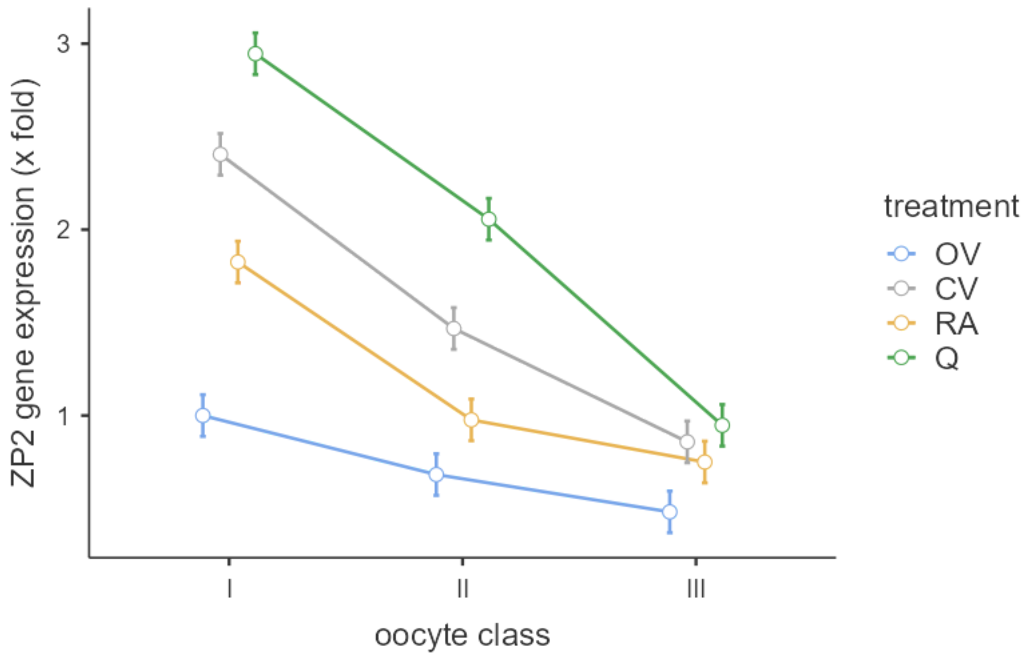

Estimated Marginal Means - oocyte class ✻ treatment

				95% Confidence Interval	
**treatment**	**oocyte class**	**Mean**	**SE**	**Lower**	**Upper**
**OV**	**I**	1.000	0.0560	0.888	1.112
	**II**	0.682	0.0560	0.570	0.794
	**III**	0.482	0.0560	0.370	0.594
**CV**	**I**	2.405	0.0560	2.293	2.517
	**II**	1.468	0.0560	1.356	1.580
	**III**	0.858	0.0560	0.746	0.970
**RA**	**I**	1.826	0.0560	1.714	1.938
	**II**	0.977	0.0560	0.865	1.089
	**III**	0.750	0.0560	0.638	0.862
**Q**	**I**	2.946	0.0560	2.834	3.058
	**II**	2.056	0.0560	1.944	2.168
	**III**	0.947	0.0560	0.835	1.059

Results

ANOVA

ANOVA - ZP3 gene expression (x fold)

	Sum of Squares	df	Mean Square	F	p
**oocyte class**	13.92	2	6.9620	222.52	<.001
**treatment**	12.03	3	4.0108	128.19	<.001
**oocyte class ✻ treatment**	1.43	6	0.2385	7.62	<.001
**Residuals**	1.88	60	0.0313		

[3]

Post Hoc Tests

Post Hoc Comparisons - oocyte class

Comparison							
**oocyte class**		**oocyte class**	**Mean Difference**	**SE**	**df**	**t**	p_{tukey}
**I**	-	**II**	0.603	0.0511	60.0	11.81	<.001
	-	**III**	1.075	0.0511	60.0	21.04	<.001
**II**	-	**III**	0.471	0.0511	60.0	9.23	<.001

Note. Comparisons are based on estimated marginal means

Post Hoc Comparisons - treatment

Comparison							
**treatment**		**treatment**	**Mean Difference**	**SE**	**df**	**t**	p_{tukey}
**OV**	-	**CV**	-0.870	0.0590	60.0	-14.75	<.001
	-	**RA**	-0.435	0.0590	60.0	-7.37	<.001
	-	**Q**	-1.057	0.0590	60.0	-17.93	<.001
**CV**	-	**RA**	0.435	0.0590	60.0	7.38	<.001
	-	**Q**	-0.187	0.0590	60.0	-3.18	0.012
**RA**	-	**Q**	-0.622	0.0590	60.0	-10.55	<.001

Note. Comparisons are based on estimated marginal means

Post Hoc Comparisons - oocyte class ✻ treatment

Comparison									
oocyte class	treatment		oocyte class	treatment	Mean Difference	SE	df	t	p_{tukey}
**I**	**OV**	-	**I**	**CV**	-1.1624	0.102	60.0	-11.382	<.001
		-	**I**	**RA**	-0.5923	0.102	60.0	-5.800	<.001
		-	**I**	**Q**	-1.4278	0.102	60.0	-13.982	<.001
		-	**II**	**OV**	0.4806	0.102	60.0	4.706	<.001
		-	**II**	**CV**	-0.5009	0.102	60.0	-4.905	<.001
		-	**II**	**RA**	-0.0349	0.102	60.0	-0.342	1.000
		-	**II**	**Q**	-0.7152	0.102	60.0	-7.004	<.001
		-	**III**	**OV**	0.5810	0.102	60.0	5.689	<.001
		-	**III**	**CV**	0.1158	0.102	60.0	1.134	0.992
		-	**III**	**RA**	0.3849	0.102	60.0	3.769	0.018
		-	**III**	**Q**	0.0339	0.102	60.0	0.332	1.000
	**CV**	-	**I**	**RA**	0.5701	0.102	60.0	5.583	<.001
		-	**I**	**Q**	-0.2654	0.102	60.0	-2.599	0.303
		-	**II**	**OV**	1.6430	0.102	60.0	16.088	<.001
		-	**II**	**CV**	0.6615	0.102	60.0	6.478	<.001
		-	**II**	**RA**	1.1275	0.102	60.0	11.041	<.001
		-	**II**	**Q**	0.4472	0.102	60.0	4.379	0.003
		-	**III**	**OV**	1.7434	0.102	60.0	17.071	<.001
		-	**III**	**CV**	1.2782	0.102	60.0	12.516	<.001
		-	**III**	**RA**	1.5473	0.102	60.0	15.151	<.001
		-	**III**	**Q**	1.1963	0.102	60.0	11.714	<.001
	**RA**	-	**I**	**Q**	-0.8355	0.102	60.0	-8.182	<.001
		-	**II**	**OV**	1.0729	0.102	60.0	10.506	<.001
		-	**II**	**CV**	0.0914	0.102	60.0	0.895	0.999
		-	**II**	**RA**	0.5574	0.102	60.0	5.458	<.001
		-	**II**	**Q**	-0.1229	0.102	60.0	-1.204	0.987
		-	**III**	**OV**	1.1733	0.102	60.0	11.489	<.001
		-	**III**	**CV**	0.7081	0.102	60.0	6.934	<.001
		-	**III**	**RA**	0.9771	0.102	60.0	9.568	<.001
		-	**III**	**Q**	0.6262	0.102	60.0	6.132	<.001
	**Q**	-	**II**	**OV**	1.9084	0.102	60.0	18.688	<.001
		-	**II**	**CV**	0.9270	0.102	60.0	9.077	<.001
		-	**II**	**RA**	1.3929	0.102	60.0	13.640	<.001
		-	**II**	**Q**	0.7126	0.102	60.0	6.978	<.001
		-	**III**	**OV**	2.0088	0.102	60.0	19.671	<.001
		-	**III**	**CV**	1.5436	0.102	60.0	15.115	<.001
		-	**III**	**RA**	1.8127	0.102	60.0	17.750	<.001
		-	**III**	**Q**	1.4617	0.102	60.0	14.314	<.001

Note. Comparisons are based on estimated marginal means

Post Hoc Comparisons - oocyte class ✻ treatment

**II**	**OV**	-	**II**	**CV**	-0.9815	0.102	60.0	-9.611	<.001
		-	**II**	**RA**	-0.5155	0.102	60.0	-5.048	<.001
		-	**II**	**Q**	-1.1958	0.102	60.0	-11.710	<.001
		-	**III**	**OV**	0.1004	0.102	60.0	0.983	0.998
		-	**III**	**CV**	-0.3648	0.102	60.0	-3.572	0.031
		-	**III**	**RA**	-0.0957	0.102	60.0	-0.937	0.998
		-	**III**	**Q**	-0.4467	0.102	60.0	-4.374	0.003
	**CV**	-	**II**	**RA**	0.4660	0.102	60.0	4.563	0.001
		-	**II**	**Q**	-0.2144	0.102	60.0	-2.099	0.625
		-	**III**	**OV**	1.0818	0.102	60.0	10.594	<.001
		-	**III**	**CV**	0.6167	0.102	60.0	6.038	<.001
		-	**III**	**RA**	0.8857	0.102	60.0	8.673	<.001
		-	**III**	**Q**	0.5348	0.102	60.0	5.237	<.001
	**RA**	-	**II**	**Q**	-0.6803	0.102	60.0	-6.662	<.001
		-	**III**	**OV**	0.6159	0.102	60.0	6.031	<.001
		-	**III**	**CV**	0.1507	0.102	60.0	1.476	0.941
		-	**III**	**RA**	0.4197	0.102	60.0	4.110	0.006
		-	**III**	**Q**	0.0688	0.102	60.0	0.674	1.000
	**Q**	-	**III**	**OV**	1.2962	0.102	60.0	12.693	<.001
		-	**III**	**CV**	0.8310	0.102	60.0	8.138	<.001
		-	**III**	**RA**	1.1001	0.102	60.0	10.772	<.001
		-	**III**	**Q**	0.7491	0.102	60.0	7.336	<.001
**III**	**OV**	-	**III**	**CV**	-0.4652	0.102	60.0	-4.555	0.001
		-	**III**	**RA**	-0.1961	0.102	60.0	-1.920	0.742
		-	**III**	**Q**	-0.5471	0.102	60.0	-5.357	<.001
	**CV**	-	**III**	**RA**	0.2691	0.102	60.0	2.635	0.284
		-	**III**	**Q**	-0.0819	0.102	60.0	-0.802	1.000
	**RA**	-	**III**	**Q**	-0.3509	0.102	60.0	-3.437	0.045

Note. Comparisons are based on estimated marginal means

[4]

Estimated Marginal Means

oocyte class ✻ treatment

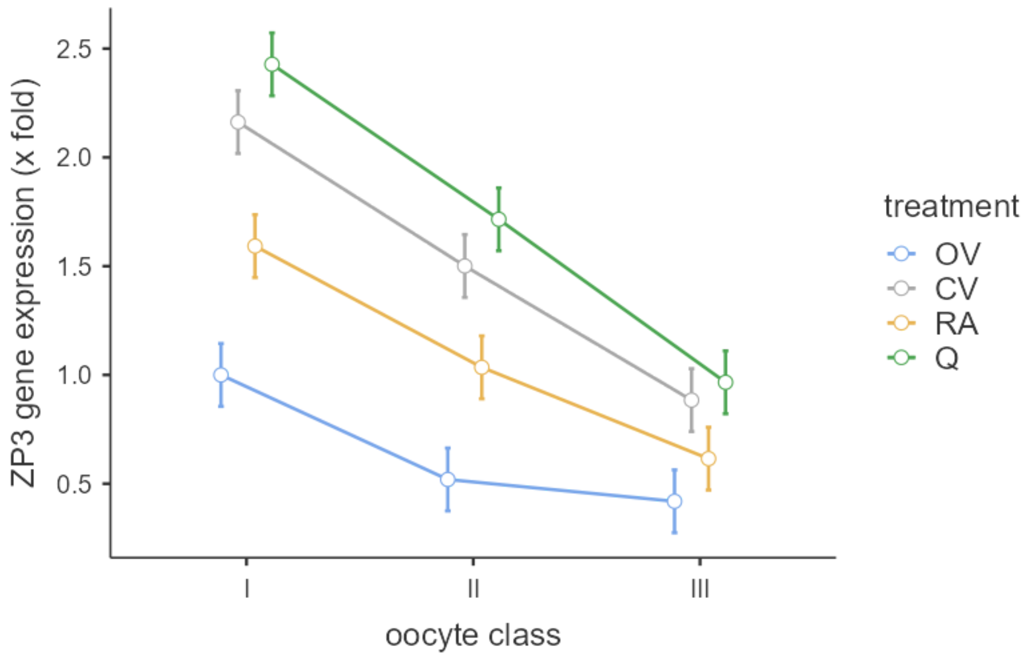

Estimated Marginal Means - oocyte class ✻ treatment

				95% Confidence Interval	
**treatment**	**oocyte class**	**Mean**	**SE**	**Lower**	**Upper**
**OV**	**I**	1.000	0.0722	0.856	1.144
	**II**	0.519	0.0722	0.375	0.664
	**III**	0.419	0.0722	0.275	0.563
**CV**	**I**	2.162	0.0722	2.018	2.307
	**II**	1.501	0.0722	1.356	1.645
	**III**	0.884	0.0722	0.740	1.029
**RA**	**I**	1.592	0.0722	1.448	1.737
	**II**	1.035	0.0722	0.890	1.179
	**III**	0.615	0.0722	0.471	0.760
**Q**	**I**	2.428	0.0722	2.283	2.572
	**II**	1.715	0.0722	1.571	1.860
	**III**	0.966	0.0722	0.822	1.111
